# Comparative Inner Morphological and Chemical Studies on *Reynoutria* Species in Korea

**DOI:** 10.3390/plants9020222

**Published:** 2020-02-09

**Authors:** Atif Ali Khan Khalil, Kazi-Marjahan Akter, Hye-Jin Kim, Woo Sung Park, Dong-Min Kang, Kyung Ah Koo, Mi-Jeong Ahn

**Affiliations:** 1 College of Pharmacy and Research Institute of Pharmaceutical Sciences, Gyeongsang National University, Jinju 52828, Korea; atif.khalil7799@gmail.com (A.A.K.K.); marjahan7silva@gmail.com (K.-M.A.); black200203@gmail.com (H.-J.K.); pws8822@gmail.com (W.S.P.); dongminkang71@gmail.com (D.-M.K.); kk408842@gmail.com (K.A.K.); 2 Department of Biological Sciences, National University of Medical Sciences, 46000 Rawalpindi, Pakistan

**Keywords:** *Reynoutria* species, inner morphological, chemical analysis, HPLC–DAD, OPLS-DA

## Abstract

*Reynoutria* species are medicinal plants that belong to the family Polygonaceae and are widely distributed in eastern Asia, North America and Europe. Although the phylogeny and morphological and anatomical studies of some species in Korea have been previously reported, there are no discriminative anatomical and chemical data available. Therefore, anatomical characterization of the leaf, stem and root, and high performance liquid chromatography–diode array detector (HPLC–DAD) analyses were carried out to assess the differences in anatomical and chemical profiles among the *Reynoutria* plants in Korea, i.e., *R. japonica*, *R. sachalinensis*, *R. forbesii* and *R. japonica* for. *elata*. The anatomical evaluation showed discriminative characteristics, such as the shape of the stomata and the stomatal index of the lower leaf surface; the ratio of the adaxial/abaxial height, the size of the vascular bundles and the frequency of druse in the midrib, petiole, and stem; and the pericycle number in the root. For the HPLC analysis, ten compounds corresponding to each major peak were isolated from *R*. *japonica* roots and their structures were identified by comprehensive spectroscopic studies. Samples collected before the flowering season showed higher contents of these ten major compounds than those collected after the flowering season. The orthogonal projections to latent structures-discrimination analysis (OPLS-DA) with the inner morphological and HPLC quantification results, clearly discriminated these plants. These results provide anatomical parameters and HPLC profiling that can be used to distinguish the four *Reynoutria* plants, which supports quality control for their precise identification.

## 1. Introduction

*Reynoutria* species (knotweed) are medicinal plants that belong to the family Polygonaceae and are widely distributed in eastern Asia, North America and Europe. The *Reynoutria* species are perennial herbs with thickened, long-branched rhizomes [1,2]. Some species have been used as food resources or traditional folk medicines for treating conditions such as neuro-cardiovascular diseases, constipation [1], inflammation, jaundice [2], hyperlipidemia and skin burns [1,2,3]. The species *R. japonica* is widely distributed in Korea, Japan, North America, Europe, and the southern part of China. The roots of this species have been reported to exhibit various beneficial biological properties, such as the inhibition of neuraminidases [4] and topoisomerases [5] and to have anti-tumor [6], anti-inflammatory [7], antibacterial [8] and antifungal activities [9]. Moreover, the roots have been used to dye rice flour, and the young stems have been used as a foodstuff [10]. *R. sachalinensis* and *R. japonica* for. *elata* are endemic plants on the Ulleung and Jeju islands of Korea, respectively [11]. *R. sachalinensis*, with its characteristic large leaves, is known to have antioxidant and anti-inflammatory activities [12,13]. The outer morphology of *R. japonica* for. *elata* is similar to *R. japonica*. The only differences are its smaller size, red flower and seeds [11]. Early works have reported many phytochemical constituents from the roots and leaves of *Reynoutria* species, including stilbenes (resveratrol and polydatin), flavonoids (rutin, apigenin, quercetin, quercitrin, isoquercitrin, hyperoside, reynoutrin and kaempferol), anthraquinones (emodin, citreorosein, physcion, fallacinol, chrysophanol, phylloquinones B and C, and anthraglycosides A and B), coumarins, essential oils, and others (lapathoside, 8-hydroxycalamenene, oleanolic acid, chlorogenic acid, protocatechuic acid, gallic acid, tachioside etc) [1,2,3,4,8,9,10,14,15,16].

Although *Reynoutria* species are used as a folk medicine, their exact botanical origin is unclear. Previous studies have reported certain *Reynoutria* species with great ambiguity in their taxonomic classification, because of variabilities in their chromosome number and morphology [17]. In addition, taxonomic problems are also compounded because interspecific hybridization is relatively common in these species [18]. There is even a report that seems to have treated *R. japonica* and *R. forbesii* as the same species [17]. Despite their outer morphological characteristics, and the fact that the genetic diversity or chemical constituents of some specimens have been previously reported, there are no previous reports of an anatomical or phytochemical study that can be applied to the decree of the botanical origin of the *Reynoutria* species [17,18,19,20,21,22,23,24,25]. Furthermore, it is very hard to discriminate crude drugs from these species precisely because they are usually sold as chopped rhizomes in the markets. Therefore, the aim of this study was to establish more precise quality control parameters based on a comprehensive inner morphological study and phytochemical profiling for the accurate pharmacognostic evaluation and differentiation of three *Reynoutria* species and one form that grows in Korea, named *R. japonica* (RJ)*, R. sachalinensis* (RS)*, R. forbesii* (RF) and *R. japonica* for. *elata* (RJE).

## 2. Results and Discussion

### 2.1. Anatomical Characteristics of the Leaf

**Figure 1:**
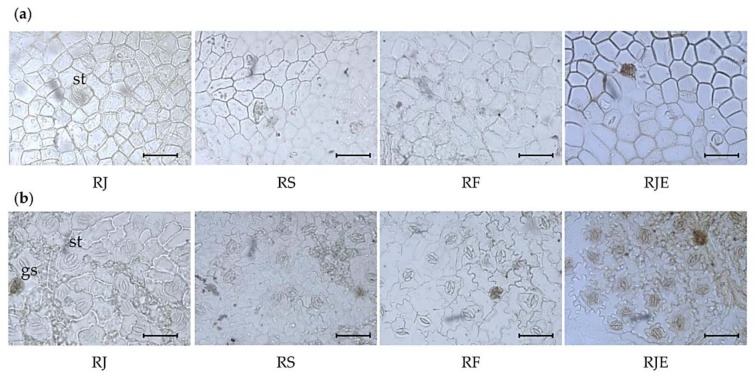
Photomicroscopic data of the leaf surface from four *Reynoutria* plants: (**a**) adaxial (above) surface of the leaf; (**b**) abaxial (lower) surface of the leaf. The black bars mean 100 µm. *st*: stomata; *gs*: glandular scale.

Leaves from the four samples showed common anatomical features, with a few differences. The adaxial epidermal cells were irregularly shaped and similar among the species (Figure 1a). All samples were amphistomatic, and numerous stomata were observed on the abaxial surface, while on the adaxial surface they were rarely seen. Two types of stomata were recognized. While the anisocytic type of stomata were common in RJ, RF, and RJE, the anomocytic type were observed in RS (Figure 1b) [20,21]. The stomatal size did not vary among the samples. The average length was between 39 and 43 µm and the average width was between 27 and 33 µm. The stomatal index was the highest, with a mean value of 19 ± 2, in RS. The frequency of stomata was lowest in RS, with a value of 19 ± 3 in mm^2^, while no significant difference was observed in the other samples. The number of glandular scales was between 3 and 5 in mm^2^, and their diameter was between 40 and 50 µm in all samples. The cell numbers in the glandular scales varied among the samples. While RJ showed only eight-celled glandular scales, four- or eight-celled glandular scales were observed in RS and RF, and four-, six-, or eight-celled glandular scales were observed in RJE (Table 1). The stomatal shape and other key anatomical aspects of RS were consistent with those in the previous report [20].

### 2.2. Anatomical Characteristics of the Midrib

**Figure 2:**
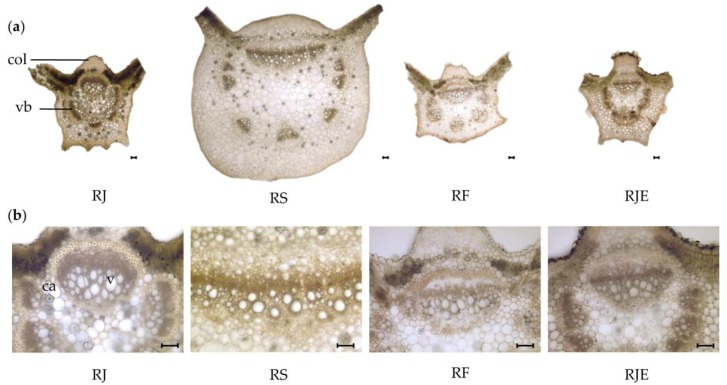
Photomicroscopic data of the midrib from four *Reynoutria* plants: (**a**) transverse section of the midrib; (**b**) Transverse section of a vascular bundle located at the center of the midrib. The black bars mean 100 µm. *ca*: druse; *col*: collenchyma cell; *vb*: vascular bundle; *v*: vessel.

A transverse section through the midrib region showed the prominent characteristics of the midribs in the lower part of the leaf (Figure 2a). The whole midrib of RS was round, and the midribs of the other three samples were pentagonal. The adaxial sides of the midrib were prominently a pyramidal cone for RJ, RF, and RJE, and a round pyramidal cone for RS. The ratio of adaxial/abaxial height was lowest in RS, with a mean value of 0.13 ± 0.02 (Table 2), while RJE showed the highest ratio of adaxial/abaxial height. The upper and lower epidermis of the midrib was composed of a single row of rectangular to square cells. A collenchyma cell layer was observed beneath the epidermis cells on both the adaxial and abaxial sides. Clusters of collenchyma cells were found on the adaxial side, while the abaxial side consisted of only one to two layers of collenchyma cells in all species. The vascular bundles were fairly prominent and were radially arranged, with a large one in the center. Moreover, a considerable difference was found in the number and size of the vascular bundles. The largest centered vascular bundle was observed in RS (565 ± 75 and 1235 ± 230 µm) (Figure 2b, Table 2). A large difference among the samples was found for the frequency of druse. The calcium oxalate crystals, with druse of a rosette shape, were most abundant in RJ, with a mean value of 36 ± 10 in mm^2^, followed by RF with 20 ± 6 in mm^2^. RJE showed the lowest frequency with 4 ± 2 6 in mm^2^. No significant difference was found in druse size or vessel diameter.

### 2.3. Anatomical Characteristics of the Petiole

**Figure 3:**
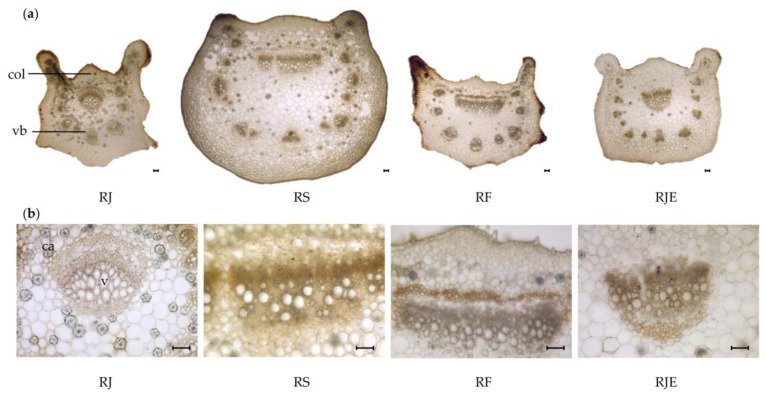
Photomicroscopic data of the petiole from four *Reynoutria* plants: (**a**) transverse section of the petiole; (**b**) transverse section of a vascular bundle located at the center of the petiole. The black bars mean 100 µm. *ca*: druse; *col*: collenchyma cell; *vb*: vascular bundle; *v*: vessel.

The transverse section of the petiole was biconvex on both ends of the adaxial portion (Figure 3a). The whole shape was somewhat round, with a pyramidal cone for RS and a pentagonal shape for RJ, RF and RJE. The upper and lower epidermis consisted of a single layer of squarish cells. Ten to fifteen layers of angular collenchyma cells were located immediately under the epidermis on the adaxial side, while on the abaxial side only one to two layers were observed. Scattered or inverted omega-shaped vascular bundles, including a big crescent-shaped bundle in the center, were seen. The ratio of adaxial/abaxial height was lowest in the RS with a mean value of 0.04 ± 0.02, while no significant difference was observed among the other samples (Table 3). In addition, the number of vascular bundles was between eleven and fifteen in all samples. The size of the vascular bundle in the center was largest in RS (Figure 3b). Meanwhile, a significant variation was found for the frequency of druse. RJ showed the highest frequency of druse with 35 ± 9 in mm^2^, followed by RF with 22 ± 6 in mm^2^. RS and RJE displayed the lowest frequency, with mean values of 10 ± 5 and 3 ± 2 in mm^2^, respectively. No significant differences were found for druse size or vessel diameter.

### 2.4. Anatomical Characteristics of the Stem

**Figure 4:**
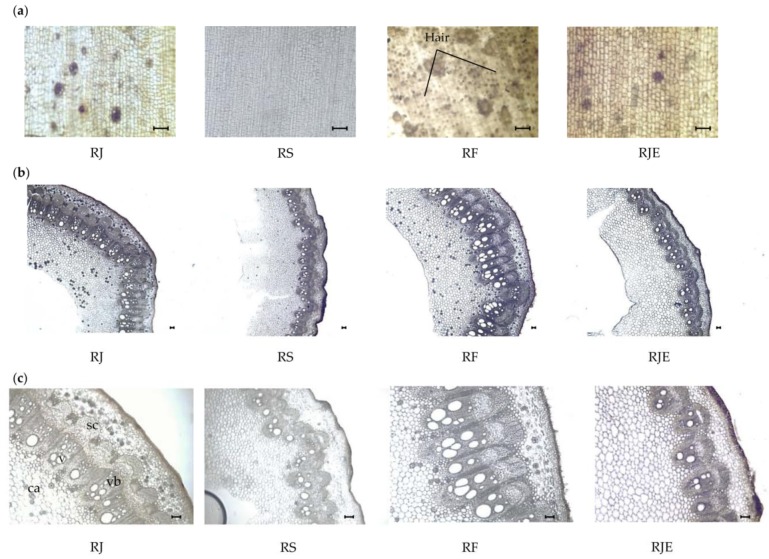
Photomicroscopic data of the stem from the four *Reynoutria* plants: (**a**) surface section of the stem; (**b**) and (**c**) transverse sections of the stem. The black bars mean 100 µm. *ca*: druse; *sc*: sclerenchyma cell; *vb*: vascular bundle; *v*: vessel.

Representative transverse and longitudinal sections were taken from the stems of the plant materials (Figure 4). The whole shape of the stem was rounded for all four samples. The stem possessed a thick and distinct epidermis of squarish or elongated cells covered with a well-defined cuticle. Hair was found only on the surface of the stem of RF with a frequency of 81 ± 10 in mm^2^ (Table 4). The cortex was angular with four to five layers of collenchyma cells. The vascular bundles were collateral and formed a continuous ring, encircling the wide pith, and were bounded by narrow medullary rays. Considerable differences were observed for the number and size of the vascular bundles. The highest number of vascular bundles was found in RS at 130 ± 10 in mm^2^, while the vascular bundles were the largest at 1000 ± 140 μm (Table 4). The druse was rosette in shape and was found in the parenchyma cells of the pith and cortex. The highest frequency of druse was noticed in RJ with 25 ± 10 in mm^2^, followed by RF (10 ± 2 in mm^2^). RS and RJE displayed the lowest frequencies, with mean values of 4 ± 3 and 2 ± 2 in mm^2^, respectively. In every vascular bundle, there were three to four woody vessels with medullary rays, and they ran radially from the cambium through the xylem. The stem was also characterized by the presence of lignified sclerenchyma cells. The lowest frequency of sclerenchyma cells was found in RS with 152 ± 32 in mm^2^. RF and RJ showed the highest frequencies with values of 264 ± 34 and 250 ± 35 in mm^2^, respectively (Table 4). Furthermore, considerable differences were observed for vessel diameters. The smallest vessel diameters were found in RS and RJE with mean values of 75 ± 20 and 75 ± 15 µm, respectively.

### 2.5. Anatomical Characteristics of the Root

**Figure 5:**
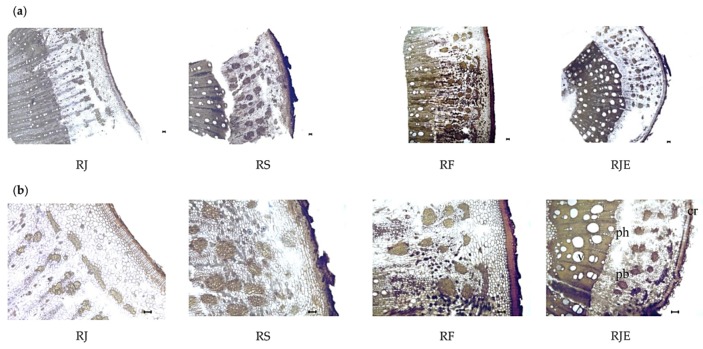
Photomicrographs of the roots from four *Reynoutria* plants: (**a**) and (**b**) transverse section of the root. The black bars mean 100 µm. *cr*: cork; *ph*: phloem; *pb*: pericycle bundle; *v*: vessel.

The roots of all samples were stout, cylindrical and dark brown. In the cross-section (Figure 5a), the roots were circular and had a well-developed cork, cortex, phloem and a central core of primary and secondary xylem. The size of the cork cells and the number of cork cell layers varied among the samples. The number of cork cell layers was the lowest in RJE at 4–6. The pericycle bundles were also well developed and formed an almost continuous ring in RJ, RF and RJE, whereas in RS, the bundles were found to be scattered. The vascular tissues were eccentric and occupied the central part of the root (Figure 5a). The highest frequency of pericycle was observed in RJE at 12 ± 3 in mm^2^, and in the other samples, the frequency of the pericycle varied from 5–8 ± 2 in mm^2^. The largest xylem vessels were observed in RS, with diameters of 139 ± 27 μm, whereas no significant differences were found among the other three samples (Table 5). Furthermore, the highest frequency of vessels was found in RJE, with 30 ± 5 in mm^2^ (Table 5).

### 2.6. HPLC–DAD Profiles of Reynoutria Plants

**Figure 6:**
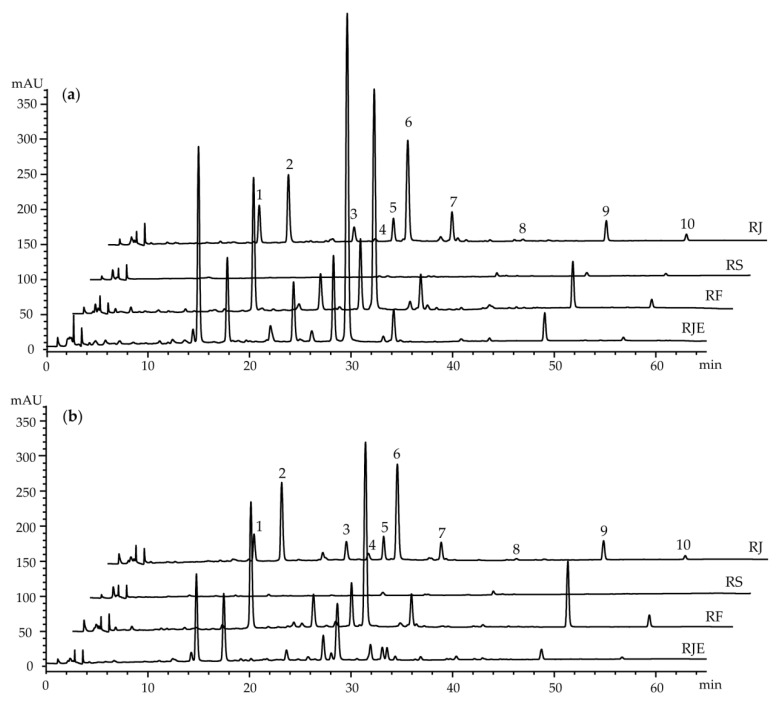
(**a**) LC chromatograms of the extracts from the roots collected before the flowering season; (**b**) LC chromatograms of the extracts from the root collected after the flowering season.

**Figure 7:**
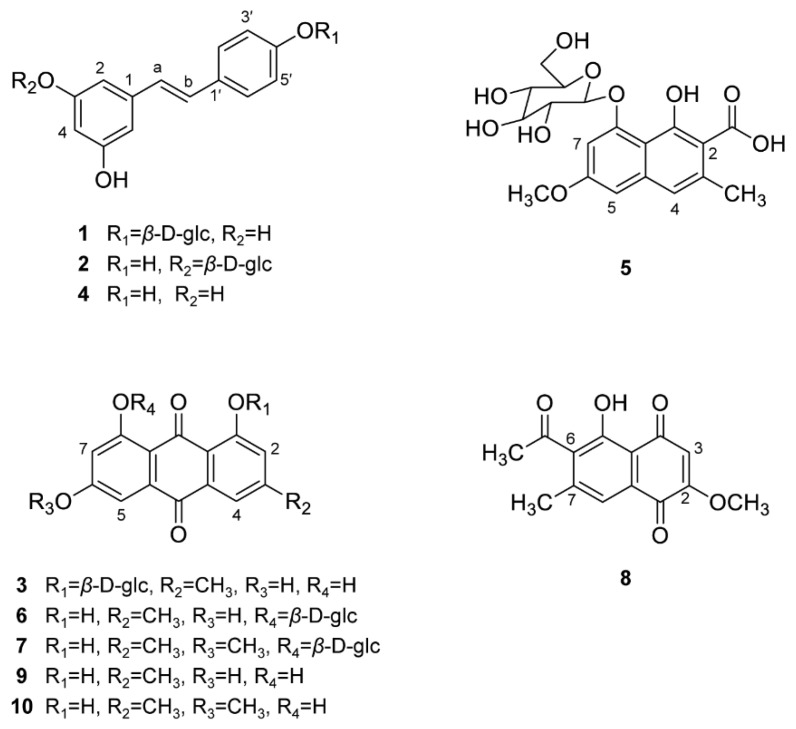
Chemical structures of peaks 1–10.

A high performance liquid chromatography–diode array detector (HPLC–DAD) analysis was employed to compare the targeted phytochemical content in the 100% methanol extracts obtained from the roots of four *Reynoutria* plants that were collected before and after flowering. Ten major peaks (**1**–**10**) were detected in the LC chromatograms (Figure 6a,b). The chemicals corresponding to each peak were determined to be three stilbenes, resveratroloside (**1**), polydatin (**2**) and *trans*-resveratrol (**4**); a naphthalene glucoside of 6-methoxy-3-methyl-1-hydroxy-2-naphthoic acid 8-*O*-*β*-D-glucopyranoside (**5**); a juglone of 2-methoxy-6-acetyl-7-methyljuglone (**8**); and five anthraquinones, emodin-1-*O*-*β*-D-glucoside (**3**), emodin-8-*O*-*β*-D-glucoside (**6**), physcion-8-*O*-*β*-D-glucoside (**7**), emodin (**9**) and physcion (**10**) (Figure 7) (see Supplementary data, Figures S1–S30). They were isolated from the root of *R. japonica* by our previously reported methods [26,27] and used as external standards for quantification. Compound **5** was isolated as a brownish powder for the first time from *R. japonica* (see Supplementary data), although this compound had been previously reported from *Fallopia multiflora* [28]. Significant differences in the content of these isolated compounds were found among the extracts. The content of compound **1** was higher in the extracts of RJE obtained before and after flowering, with values of 75.1 ± 4.26 and 34.5 ± 0.67 mg/g dry weight (DW), respectively, than in the extracts from RJ, with values of 19.6 ± 2.15 and 11.2 ± 0.26 mg/g DW, respectively. However, Compound **1** was not detected in RS or RF. The highest content of compound **2** was found in the extracts of RF obtained before and after flowering, with values of 24.7 ± 0.40 and 21.8 ± 0.60 mg/g DW, respectively. The content was lowest in RS, with mean values of 0.05 ± 0.01 and 0.29 ± 0.01 mg/g DW, respectively. Compounds **3**, **4**, and **5** were not detected in RS (Table 6). However, the contents of compounds **3** and **4** were the highest in RJE, with mean values of 2.30 ± 0.08 and 2.04 ± 0.18 mg/g DW in the extracts obtained before flowering. For compound **5**, RF showed the highest content of 3.72 ± 0.21 mg/g DW among the samples. The content of compound **6** was the highest in the extract of RJE obtained before flowering, with a value of 16.8 ± 0.23 mg/g DW. In addition, the content of compound 7 was the lowest in RS. Furthermore, compound **8** was not detected in RS and the content ranged from 0.04–0.18 mg/g DW in the before- and after-flowering extracts. The highest contents of compounds **9** and **10** were found in the extracts of RF obtained before and after flowering (Table 6). In summary, compounds **1**, **3**, **4**, **5** and **8** were absent in the extracts of RS obtained before and after flowering, and the contents of the detected compounds in RS were lowest among the samples. In addition, the before-flowering extracts displayed higher contents of the ten major compounds than the after-flowering extracts. The result that stilbenes and anthraquinones are major secondary metabolites of RJ is consistent with the previous data [23]. The contents of polydatin and resveratrol in dried rhizomes of RJ was similar to those of a previous report [24]. However, phenylpropanoid glucosides were not determined in RS by our analytical method, while there is a report that these compounds were detected in RS by LC–MS analysis [23]. This discrepancy resulted from different analytical methods.

### 2.7. OPLS-DA Multivariate Statistical Analysis

**Figure 8:**
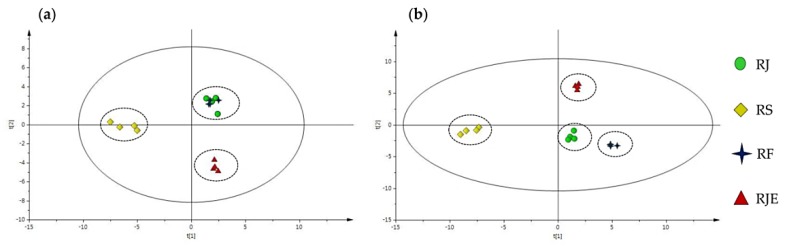
(**a**) Score plots by OPLS-DA with anatomical characteristics only; (**b**) OPLS-DA with anatomical characteristics combined with the HPLC profile data for the four *Reynoutria* plants.

Orthogonal projections to latent structures-discrimination analysis (OPLS-DA) multivariate statistical analyses were applied to the anatomical characteristics and HPLC profile data to examine whether the three species and one form can be differentiated using these data (Figure 8a,b).

RJ and RF were similar in the ratio of adaxial/abaxial height, and in the number and size of vascular bundles in the midrib and petiole. However, RJE was significantly discrete from the two plants RJ and RF, although these three plants showed similar stomatal indexes, stomatal shapes and stomatal numbers. Their frequencies of druse and hair make them slightly discriminated from each other. RS was found to be significantly different from the other three plants in factors such as the stomatal index, number and size of the vascular bundle, frequency of druse, ratio of adaxial/abaxial height and the diameter of vessels in the root (Figure 8a). As a result, using all of the anatomical characteristics, the OPLS-DA model failed to efficiently discriminate each group (Figure 8a). However, the OPLS-DA combined with the HPLC profiles more clearly discriminated and classified the four *Reynoutria* plants (Figure 8b).

## 3. Materials and Methods 

### 3.1. Plant Materials and Reagents

Three *Reynoutria* species and one form were collected from four different regions of Jinju, Seoul, Jeju and Ulleung-do of Korea between August and October in 2016–2018. The samples were identified by Dr. Mi-Jeong Ahn, College of Pharmacy, Gyeongsang National University, and the voucher specimens (PGSC210–PGSC213) were deposited in the herbarium of the College of Pharmacy, Gyeongsang National University (Table 7). Two endemic plants, *R. sachalinensis* and *R. japonica* for. *elata*, were collected from their unique islands and were planted and acclimatized in Jinju (the mainland of Korea) for two or three years. *R. forbesii* also was planted and acclimatized in Jinju, like the former two species, after collection in Seoul.

Then, 50% glycerin (Junsei Chemicals Co., Ltd., Tokyo, Japan) was used to make a specimen for anatomical study. Methanol (Daejung Chemicals & Metals Co., Ltd., Shiheung, Korea) was used for the extraction of samples. The LC analysis was performed using acetonitrile and water (Fisher Scientific, Korea Ltd., Seoul, Korea). NMR solvents were purchased from Cambridge Isotope laboratories, Inc., (Andover, MA, USA).

### 3.2. Inner Morphological Study

Same-aged and well-acclimatized samples (leaf surface, midrib, petiole, stem and root) were obtained from the three *Reynoutria* species and one form. More than six intra-species samples (more than two individual plants per geographical coordinate), at three different sets of geographical coordinates of two rather different places with varying environments, were used for the anatomical metrics. Four to six slices were taken from the lower part of the midrib, and the medium part of the petiole and the stem. For the root, a slice was taken from a quarter part of the root, and the starch granules were removed under sonication for one minute, three times each. In addition, the upper and lower surfaces of the leaves were peeled off for observation. Transverse as well as longitudinal sections were taken using a hand slicer or a microslicer. The samples were placed on glass slides and mounted in glycerinated water (50%). All of the samples were observed under a photomicroscope, and the microphotographs were taken using image processing software (IMT i-Soultion inc., BC, Canada) connected to a video camera (PixeLINK, ON, Canada) for image capture.

The transverse sections were used to count the frequency of druse and the surface sections were used to determine the frequency of hair. The 1 × 1 mm^2^ range was chosen to count the number of stomata and the glandular scales on the lower leaf surface; the frequency of druse for the midrib, petiole and stem; and the number of pericycle bundles and frequency of the vessels in the roots. More than three specimens were used to obtain representative characteristics for each plant.

### 3.3. Preparation of Extracts

The dried root samples were ground in a blender and then one gram was taken from each sample. Fifty millimeters of methanol were added, and the mixtures were extracted under sonication for 60 min, three times each. The extract was filtered through filter paper (Whatman No. 2, Toyo Roshi Kaisha, Ltd. Hannan, Japan). The final volume was adjusted to 50 mL with methanol. Before HPLC analysis, the extracts were filtered through a 0.45 μm PTFE syringe filter (Whatman, New York, NY, USA).

### 3.4. HPLC–DAD Analysis

The HPLC–DAD analysis was performed on an Agilent 1260 series HPLC system equipped with an autosampler, a column oven, a binary pump and a degasser (Agilent Technologies, Palo Alto, CA, USA). An aliquot (10 μL) of the sample solution was directly injected onto a J’sphere C18 column (4.6 × 150 mm, 4 μm, YMC Co. Ltd., Kyoto, Japan) with a compatible guard column. The gradient elution using an acetonitrile-water (*v/v*) solvent system was as follows: 10% acetonitrile to 40% for the first 35 min, 40% acetonitrile to 100% for a further 25 min, and 100% for another 5 min. A conditioning phase was then used to return the column to its initial state for 5 min. The flow rate was 1.0 mL/min, and the column temperature was 30 °C. The eluent was detected at 254 nm with a DAD (diode array detector). Chemstation software (Agilent Technologies) was used to operate this LC–DAD system.

### 3.5. Statistical Analysis

Orthogonal projections to latent structure-discriminant analysis (OPLS-DA) multivariate analysis was performed with SIMCA 13 statistical software (Umetrics, Sweden). All of the anatomical characteristic data from the leaf, midrib, petiole, stem, and root of each sample and the LC profile data were used for OPLS-DA. All data were expressed as the mean ± standard deviation (SD), and one-way analysis of variance (ANOVA) was used for the statistical analysis using SPSS (Statistical Package for the Social Sciences) software (version 16). Values of *p* < 0.05 were considered statistically significant.

## 4. Conclusions

In conclusion, there were considerable differences in the anatomical characteristics and chemical profiling of three *Reynoutria* species and one form. These results indicated significant differences in their anatomical characteristics, including the stomatal number and shape on the lower leaf surface; the number and size of the vascular bundles, and the frequency of druse in the midrib, petiole, and stem; and the pericycle number, and the size of vessels and cork cells in the root. HPLC chromatograms displayed considerable differences in the contents of ten major chemical constituents among the samples, especially for resveratroloside, polydatin and emodin-8-*O*-*β*-D-glucoside. Furthermore, resveratroloside was not found in *R*. *forbesii* and *R*. *sachalinensis,* which can be used as an important marker for chemotaxonomy. In addition, the OPLS-DA multivariate statistical analysis of anatomical characteristics and HPLC profile data may be useful for the differentiation of *Reynoutria* plants.

## Figures and Tables

**Table 1:** Anatomical characteristics of the lower leaf surface of *Reynoutria* plants.

Parameters	RJ	RS	RF	RJE
Stomatal shape	anisocytic	anomocytic	Anisocytic	Anisocytic
Stomatal index	16 ± 1 ^b^	19 ± 2 ^a^	17 ± 1 ^b^	15 ± 1 ^b^
Frequency of stomata (in mm^2^)	26 ± 4 ^a^	19 ± 3 ^b^	29 ± 5 ^a^	29 ± 4 ^a^
Cell number in glandular scale	eight	four or eight	four or eight	four, six or eight

Values were expressed as the mean ± SD (*n >* 6). The different upper letters in the same line indicate significant differences (*p* < 0.05) among the samples.

**Table 2:** Anatomical characteristics of the midrib from four *Reynoutria* plants.

Parameters	RJ	RS	RF	RJE
Ratio of adaxial/abaxial height	0.21 ± 0.02 ^b^	0.13 ± 0.02 ^c^	0.22 ± 0.02 ^b^	0.29 ± 0.02 ^a^
Number of vascular bundles	7 ± 1 ^b^	10 ± 1 ^a^	7 ± 1 ^b^	7 ± 1 ^b^
Length of the vascular bundle in the center (µm)	405 ± 60 ^b^	565 ± 75 ^a^	350 ± 45 ^b^	400 ± 70 ^b^
Width of the vascular bundle in the center (µm)	525 ± 130 ^b^	1235 ± 230 ^a^	580 ± 100 ^b^	570 ± 95 ^b^
Frequency of druse (in mm^2^)	36 ± 10 ^a^	12 ± 4 ^c^	20 ± 6 ^b^	4 ± 2 ^d^

Values were expressed as the mean ± SD (*n >* 6). The different upper letters in the same line indicate significant differences (*p* < 0.05) among the samples.

**Table 3:** Anatomical characteristics of the petiole from four *Reynoutria* plants.

Parameters	RJ	RS	RF	RJE
Ratio of adaxial/abaxial height	0.10 ± 0.04 ^a^	0.04 ± 0.02 ^b^	0.11 ± 0.03 ^a^	0.12 ± 0.03 ^a^
Number of vascular bundles	13 ± 2 ^a^	15 ± 2 ^a^	11 ± 1 ^b^	13 ± 2 ^a^
Length of the vascular bundle in the center (µm)	535 ± 50 ^b^	700 ± 40 ^a^	460 ± 40 ^b^	455 ± 40 ^b^
Width of the vascular bundle in the center (µm)	635 ± 70 ^c^	1335 ± 130 ^a^	800 ± 100 ^b^	600 ± 85 ^c^
Frequency of druse (in mm^2^)	35 ± 9 ^a^	10 ± 5 ^c^	22 ± 6 ^b^	3 ± 2 ^d^

Values were expressed as mean ± SD (*n >* 6). The different upper letters in the same line indicate significant differences (*p* < 0.05) among the samples.

**Table 4:** Anatomical characteristics of the stem from four *Reynoutria* plants.

Parameters	RJ	RS	RF	RJE
Frequency of hair (in mm^2^)	0	0	81 ± 10 ^a^	0
Number of vascular bundles	95 ± 20 ^b^	130 ± 10 ^a^	110 ± 10 ^ab^	65 ± 10 ^c^
Diameter of vascular bundles (µm)	843 ± 175 ^b^	506 ± 101 ^c^	1000 ± 140 ^a^	536 ± 108 ^bc^
Frequency of druse (in mm^2^)	25 ± 10 ^a^	4 ± 3 ^c^	10 ± 2 ^b^	2 ± 2 ^c^
Frequency of sclerenchyma cells (in mm²)	250 ± 35 ^a^	152 ± 32 ^c^	264 ± 34 ^a^	192 ± 33 ^b^
Vessel diameter (µm)	117 ± 10 ^b^	75 ± 20 ^c^	140 ± 35 ^a^	75 ± 15 ^c^

Values were expressed as the mean ± SD (*n >* 6). The different upper letters in the same line indicate significant differences (*p* < 0.05) among the samples.

**Table 5:** Anatomical characteristics of the root from four *Reynoutria* plants.

Parameters	RJ	RS	RF	RJE
Size of cork cell (length × width, µm)	20 ± 3 ^a^ × 45 ± 5 ^b^	10 ± 2 ^c^ × 55 ± 5 ^a^	15 ± 2 ^b^ × 45 ± 10 ^b^	10 ± 1 ^c^ × 40 ± 10 ^b^
Number of cork cell layers	9–12 ^a^	6–8 ^b^	7–10 ^a^	4–6 ^c^
Frequency of pericycle bundles (in mm^2^)	8 ± 2 ^b^	7 ± 1 ^b^	5 ± 2 ^b^	12 ± 3 ^a^
Vessel diameter (µm)	95 ± 35 ^b^	139 ± 27 ^a^	113 ± 19 ^b^	111 ± 20 ^b^
Frequency of vessels (in mm^2^)	23 ± 5 ^b^	15 ± 2 ^c^	15 ± 2 ^c^	30 ± 5 ^a^

Values were expressed as the mean ± SD (*n >* 6). The different upper letters in the same line indicate significant differences (*p* < 0.05) among the samples.

**Table 6:** Contents of ten compounds (**1**–**10**) in the roots of four *Reynoutria* samples before and after flowering.

Compounds	Season	RJ	RS	RF	RJE
Resveratroloside (**1**)	Before	19.6 ± 2.15 ^b^	ND	ND	75.1 ± 4.26 ^a^
	After	11.2 ± 0.26 ^b^	ND	ND	34.5 ± 0.67 ^a^
Polydatin (picein) (**2**)	Before	11.9 ± 0.64 ^b^	0.05 ± 0.01 ^c^	24.7 ± 0.40 ^a^	13.0 ± 0.77 ^b^
	After	13.4 ± 0.41 ^b^	0.29 ± 0.01 ^d^	21.8 ± 0.60 ^a^	11.0 ± 0.21 ^c^
Emodin-1-*O*-*β*-D-glucoside (**3**)	Before	0.54 ± 0.02 ^c^	ND	1.28 ± 0.03 ^b^	2.30 ± 0.08 ^a^
	After	0.75 ± 0.05 ^b^	ND	1.17 ± 0.10 ^a^	0.37 ± 0.07 ^c^
*trans*-Resveratrol (**4**)	Before	0.69 ± 0.03 ^b^	ND	0.70 ± 0.05 ^b^	2.04 ± 0.18 ^a^
	After	1.69 ± 0.27 ^a^	ND	1.34 ± 0.08 ^b^	0.76 ± 0.05 ^c^
Naphthalene glucoside (**5**)	Before	1.22 ± 0.06 ^c^	ND	3.72 ± 0.21 ^a^	2.90 ± 0.08 ^b^
	After	1.05 ± 0.06 ^b^	ND	2.17 ± 0.10 ^a^	1.15 ± 0.20 ^b^
Emodin-8-*O*-*β*-D-glucoside (**6**)	Before	5.23 ± 0.28 ^c^	0.10 ± 0.01 ^d^	13.1 ± 0.49 ^b^	16.8 ± 0.23 ^a^
	After	5.63 ± 0.11 ^b^	0.13 ± 0.01 ^d^	12.3 ± 0.69 ^a^	3.35 ± 0.63 ^c^
Physcion-8-*O*-*β*-D-glucoside (**7**)	Before	1.30 ± 0.04 ^c^	0.03 ± 0.00 ^d^	1.91 ± 0.08 ^a^	1.50 ± 0.14 ^b^
	After	0.81 ± 0.03 ^b^	0.02 ± 0.00 ^d^	1.66 ± 0.06 ^a^	0.64 ± 0.05 ^c^
2-methoxy-6-acetyl-7-methyljuglone (**8**)	Before	0.04 ± 0.00 ^c^	ND	0.18 ± 0.01 ^a^	0.10 ± 0.00 ^b^
	After	0.04 ± 0.01 ^b^	ND	0.12 ± 0.02 ^a^	0.12 ± 0.01 ^a^
Emodin (**9**)	Before	0.70 ± 0.06 ^b^	0.11 ± 0.01 ^c^	1.30 ± 0.09 ^a^	0.71 ± 0.04 ^b^
	After	0.58 ± 0.05 ^b^	ND	2.09 ± 0.03 ^a^	0.34 ± 0.03 ^c^
Physcion (**10**)	Before	0.18 ± 0.02 ^a^	0.04 ± 0.01 ^b^	0.21 ± 0.03 ^a^	0.05 ± 0.01 ^b^
	After	0.07 ± 0.01 ^b^	ND	0.24 ± 0.02 ^a^	0.05 ± 0.01 ^b^

Data were expressed as mean ± SD (mg/g dry weight) (*n* = 3). The different upper letters in the same line indicate significant differences (*p* < 0.05) among the samples.

**Table 7:** A list of *Reynoutria* plants collected from Korea.

Botanical Name	Collection Place (Latitude, Longitude)	Collection Year	Voucher Specimens No.	Abbre- Viations
*R. japonica* Houtt.	Sancheong (35°18′00.4″ N, 127°58′20.9″ E) Jinju (35°10′19.8″ N, 128°05′48.6″ E) (35°13′00.6″ N, 128°01′39.5″ E)	2016-2018	PGSC-210	RJ
*R. sachalinensis* (F.Schmidt) Nakai	Ulleung island (37°27′51.4″ N, 130°52′20.4″ E) (37°27′53.8″ N, 130°52′21.6″ E) Jinju (35°08′55.1″ N, 128°05′49.6″ E)	2016-2018	PGSC-211	RS
*R. forbesii* (Hance) T. Yamaz	Seoul (37°42′46.3″ N, 126°49′07.3″ E) Jinju (35°08′55.3″ N, 128°05′49.4″ E) (35°12′55.2″ N, 128°04′08.7″ E)	2016-2018	PGSC-212	RF
*R. japonica* for. *elata*	Jeju island (33°20′49.9″ N, 126°29′47.0″ E) (33°20′41.4″ N, 126°29′40.9″ E) Jinju (35°08′55.6″ N, 128°05′49.6″ E)	2016-2018	PGSC-213	RJE

## Supplementary Materials

The following are available online at https://www.mdpi.com/2223-7747/9/2/222/s1, Figure S1: The FAB-MS spectrum of compound **1**, Figure S2: The ^1^H-NMR spectrum of compound **1**, Figure S3: The ^13^C-NMR spectrum of compound **1**, Figure S4: The FAB-MS spectrum of compound **2**, Figure S5: The ^1^H-NMR spectrum of compound **2**, Figure S6: The ^13^C-NMR spectrum of compound **2**, Figure S7: The FAB-MS spectrum of compound **3**, Figure S8: The ^1^H-NMR spectrum of compound **3**, Figure S9: The ^13^C-NMR spectrum of compound **3**, Figure S10: The EI-MS spectrum of compound **4**, Figure S11: The ^1^H-NMR spectrum of compound **4**, Figure S12: The ^13^C-NMR spectrum of compound **4**, Figure S13: The FAB-MS spectrum of compound **5**, Figure S14: The ^1^H-NMR spectrum of compound **5**, Figure S15: The ^13^C-NMR spectrum of compound **5**, Figure S16: The FAB-MS spectrum of compound **6**, Figure S17: The ^1^H-NMR spectrum of compound **6**, Figure S18: The ^13^C-NMR spectrum of compound **6**, Figure S19: The ESI-MS spectrum of compound **7**, Figure S20: The ^1^H-NMR spectrum of compound **7**, Figure S21: The ^13^C-NMR spectrum of compound **7**, Figure S22: The EI-MS spectrum of compound **8**, Figure S23: The ^1^H-NMR spectrum of compound **8**, Figure S24: The ^13^C-NMR spectrum of compound **8**, Figure S25: The EI-MS spectrum of compound **9**, Figure S26: The ^1^H-NMR spectrum of compound **9**, Figure S27: The ^13^C-NMR spectrum of compound **9**, Figure S28: The EI-MS spectrum of compound **10**, Figure S29: The ^1^H-NMR spectrum of compound **10**, Figure S30: The ^13^C-NMR spectrum of compound **10**; ^1^H and ^13^C NMR assign data of isolated compounds.
