# Supplementary material for: Comparative Inner Morphological and Chemical Studies on Reynoutria Species in Korea
Source: Plants (Basel). 2020 Feb 9;9(2):222. doi: 10.3390/plants9020222 (PMC7076709; doi:10.3390/plants9020222)
Supplement: Supplementary file 1 [file plants-09-00222-s001.pdf]

Supplementary Data

Comparative inner morphological and chemical studies on *Reynoutria* species in Korea

Atif Ali Khan Khalil†, Kazi-Marjahan Akter†, Hye-Jin Kim†, Woo Sang Park, Dong-Min Kang, Kyung Ah Koo and Mi-Jeong Ahn*

Figure S1. The FAB-MS spectrum of compound **1**
Figure S2. The ^{1}H-NMR spectrum of compound **1**
Figure S3. The ^{13}C-NMR spectrum of compound **1**
Figure S4. The FAB-MS spectrum of compound **2**
Figure S5. The ^{1}H-NMR spectrum of compound **2**
Figure S6. The ^{13}C-NMR spectrum of compound **2**
Figure S7. The FAB-MS spectrum of compound **3**
Figure S8. The ^{1}H-NMR spectrum of compound **3**
Figure S9. The ^{13}C-NMR spectrum of compound **3**
Figure S10. The EI-MS spectrum of compound **4**
Figure S11. The ^{1}H-NMR spectrum of compound **4**
Figure S12. The ^{13}C-NMR spectrum of compound **4**
Figure S13. The FAB-MS spectrum of compound **5**
Figure S14. The ^{1}H-NMR spectrum of compound **5**
Figure S15. The ^{13}C-NMR spectrum of compound **5**
Figure S16. The FAB-MS spectrum of compound **6**
Figure S17. The ^{1}H-NMR spectrum of compound **6**
Figure S18. The ^{13}C-NMR spectrum of compound **6**
Figure S19. The ESI-MS spectrum of compound **7**
Figure S20. The ^{1}H-NMR spectrum of compound **7**
Figure S21. The ^{13}C-NMR spectrum of compound **7**
Figure S22. The EI-MS spectrum of compound **8**
Figure S23. The ^{1}H-NMR spectrum of compound **8**
Figure S24. The ^{13}C-NMR spectrum of compound **8**
Figure S25. The EI-MS spectrum of compound **9**
Figure S26. The ^{1}H-NMR spectrum of compound **9**
Figure S27. The ^{13}C-NMR spectrum of compound **9**
Figure S28. The EI-MS spectrum of compound **10**
Figure S29. The ^{1}H-NMR spectrum of compound **10**
Figure S30. The ^{13}C-NMR spectrum of compound **10**
^{1}H and ^{13}C NMR assign data of isolated compounds

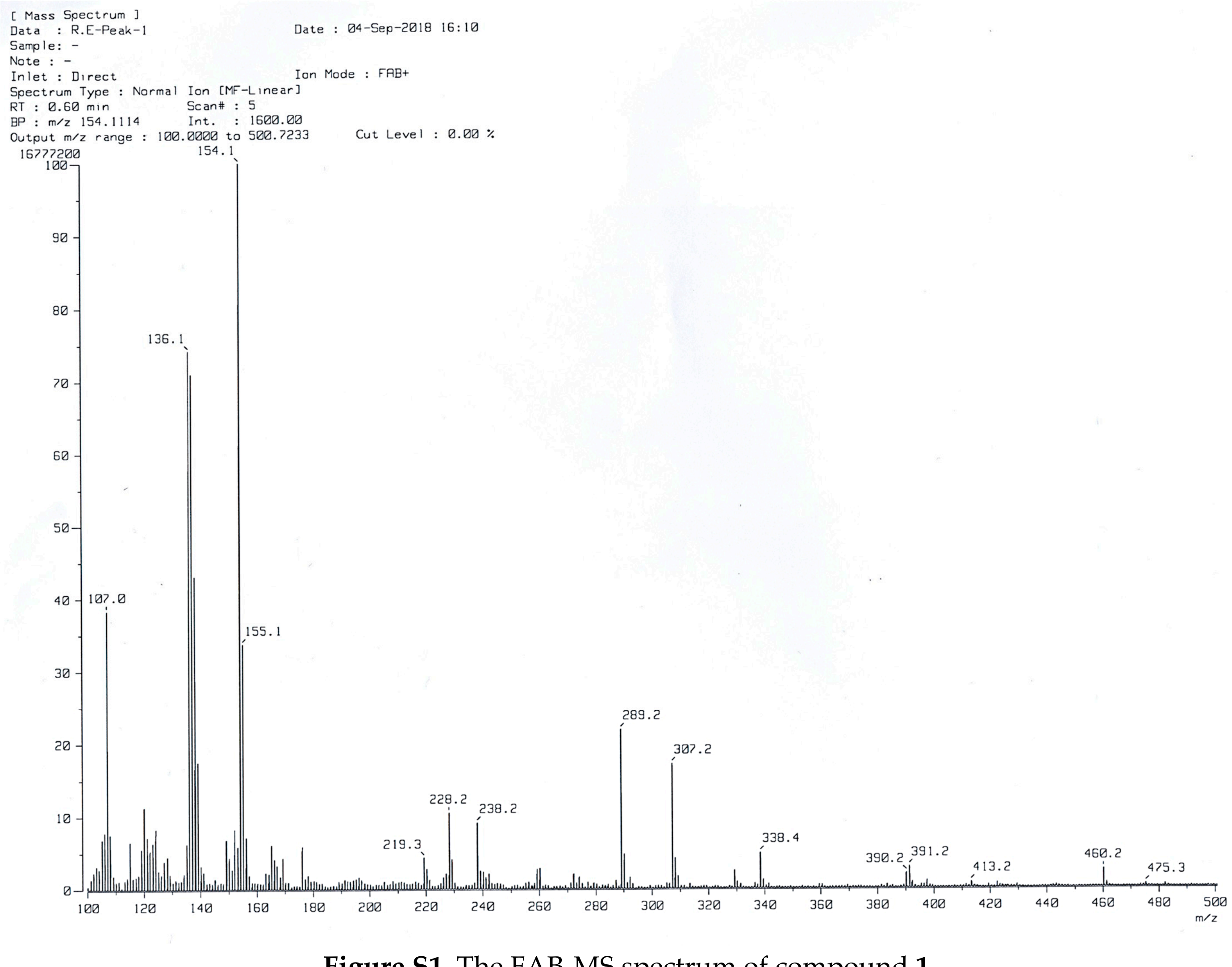

Figure S1. The FAB-MS spectrum of compound **1**

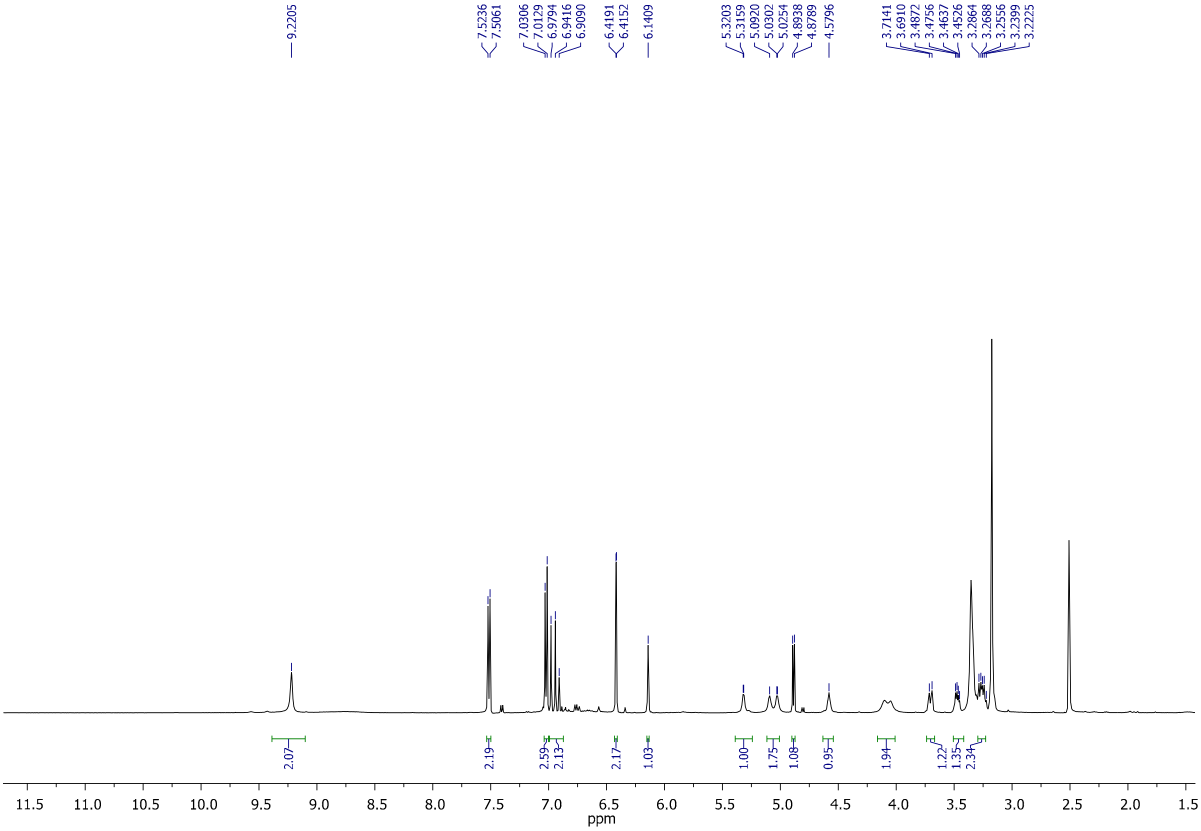

Figure S2. The ^{1}H-NMR spectrum of compound **1** (DMSO-d_6, 500 MHz)

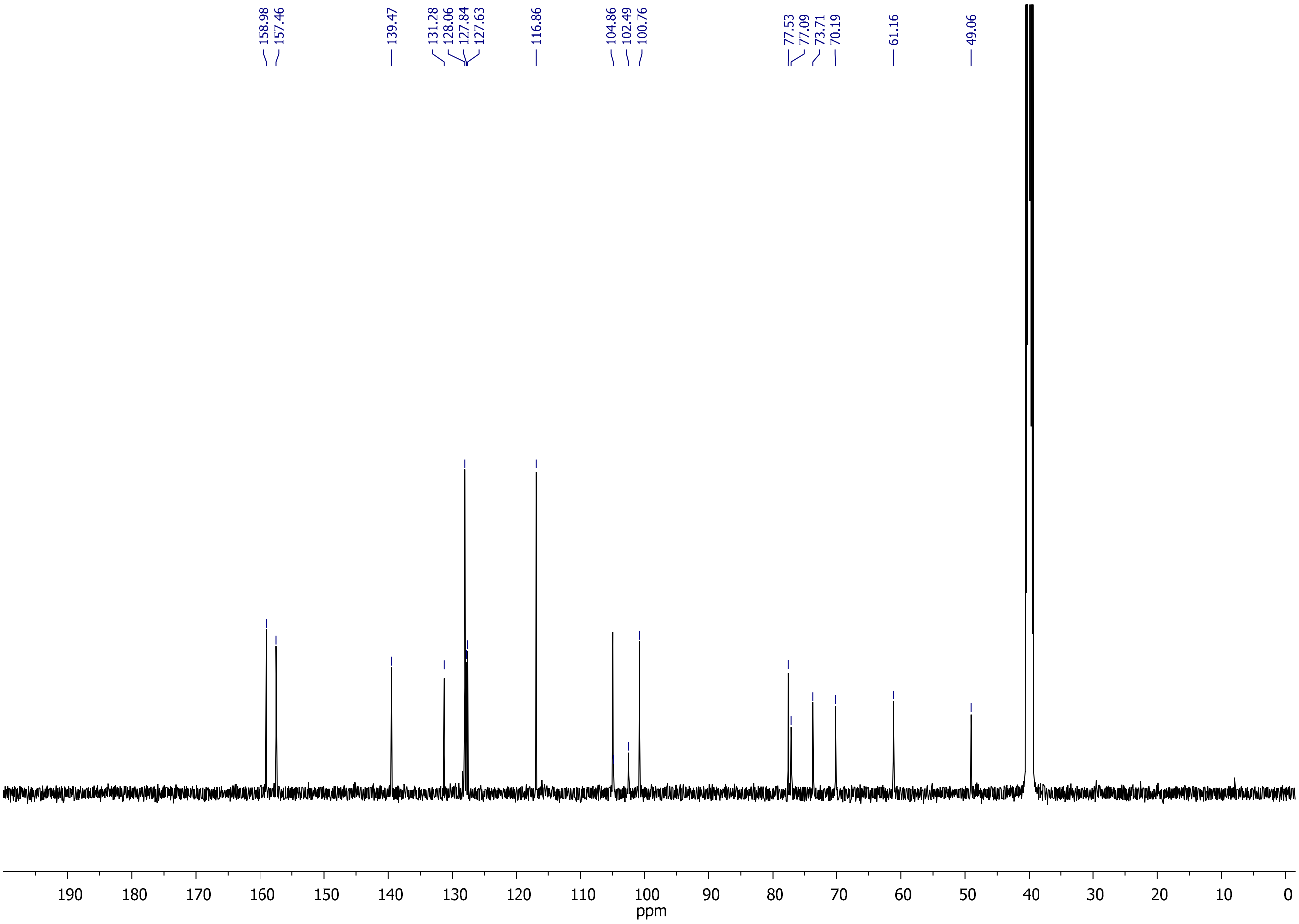

Figure S3. The ^{13}C-NMR spectrum of compound **1** (DMSO-d_6, 125 MHz)

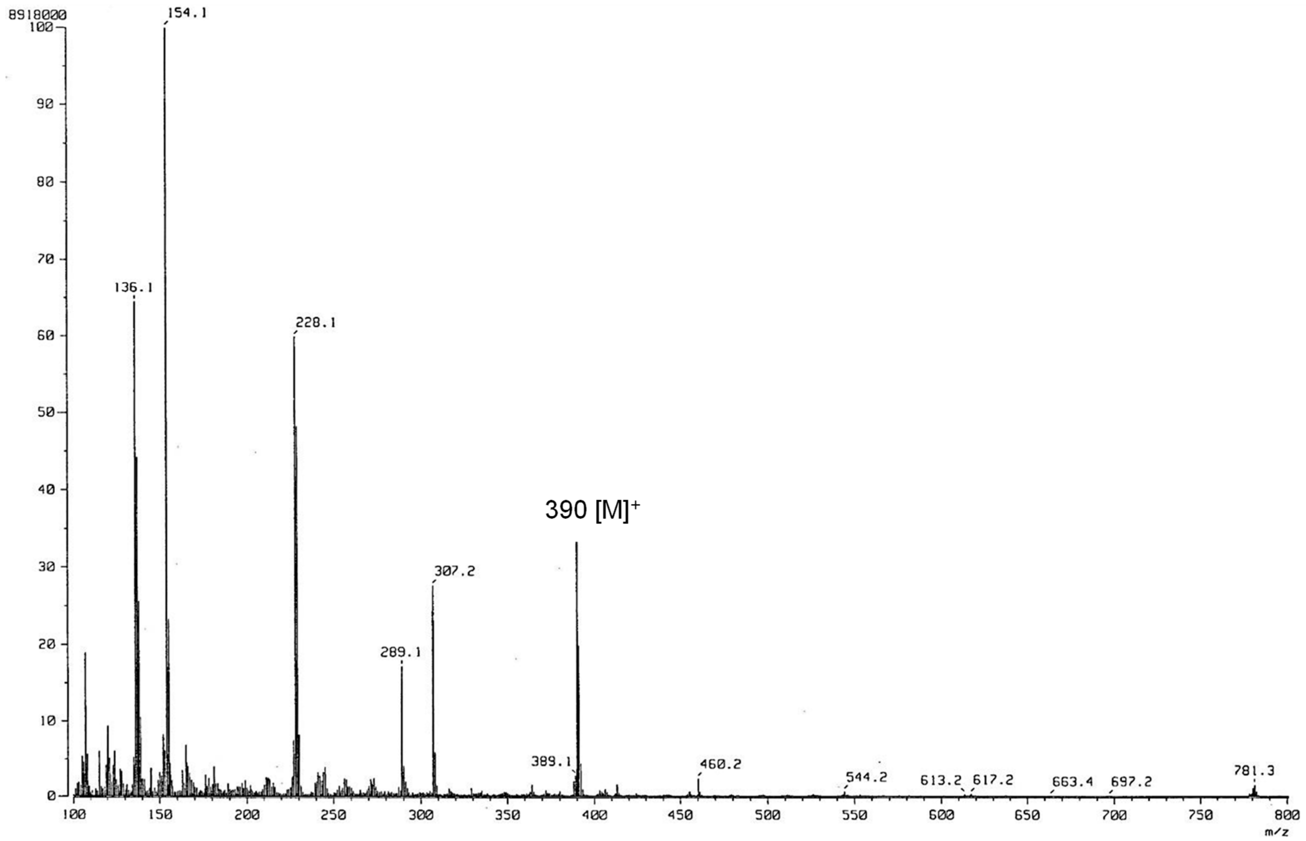

Figure S4. The FAB-MS spectrum of compound **2**

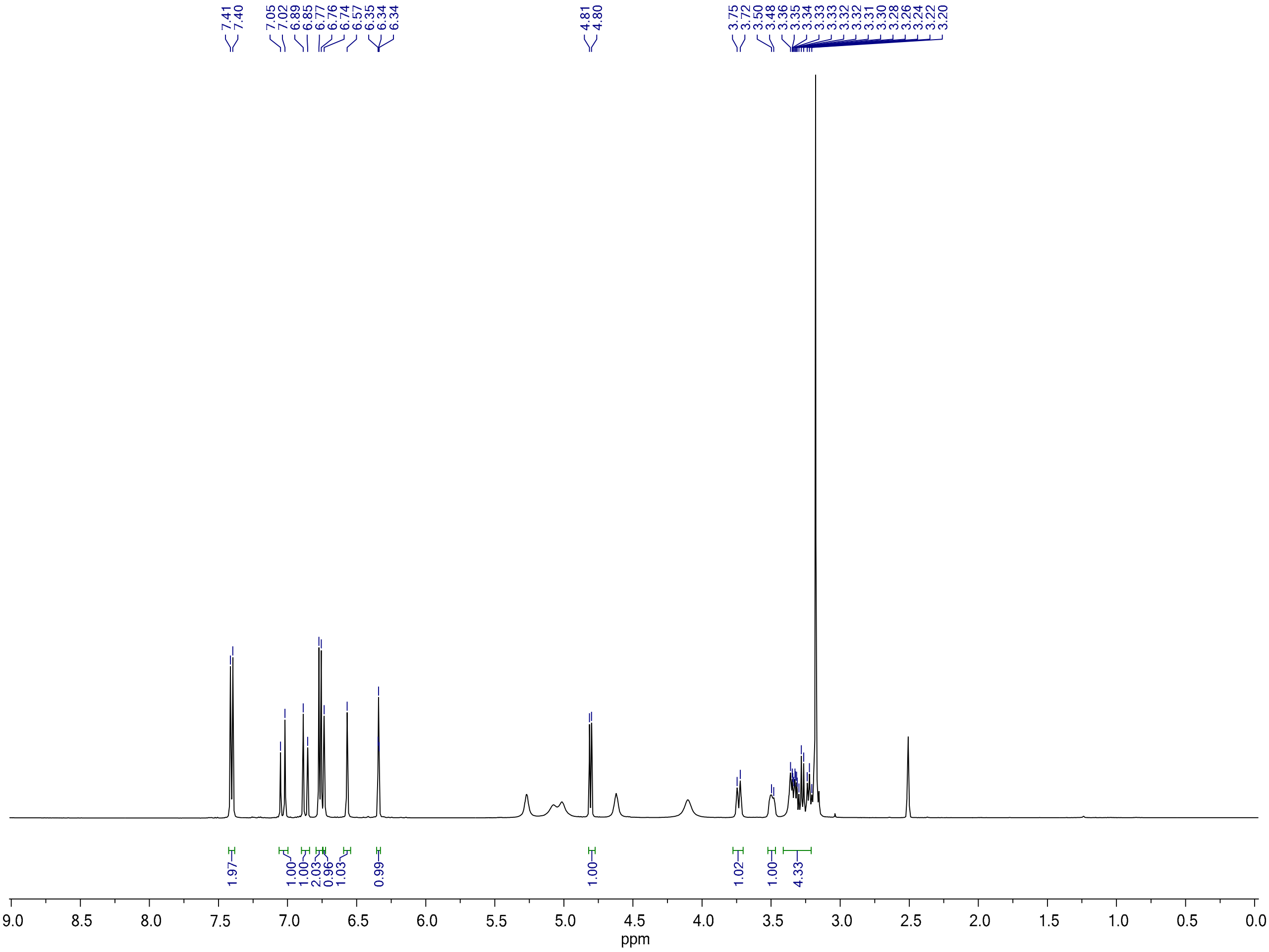

Figure S5. The ^{1}H-NMR spectrum of compound **2** (DMSO-d_6, 500 MHz)

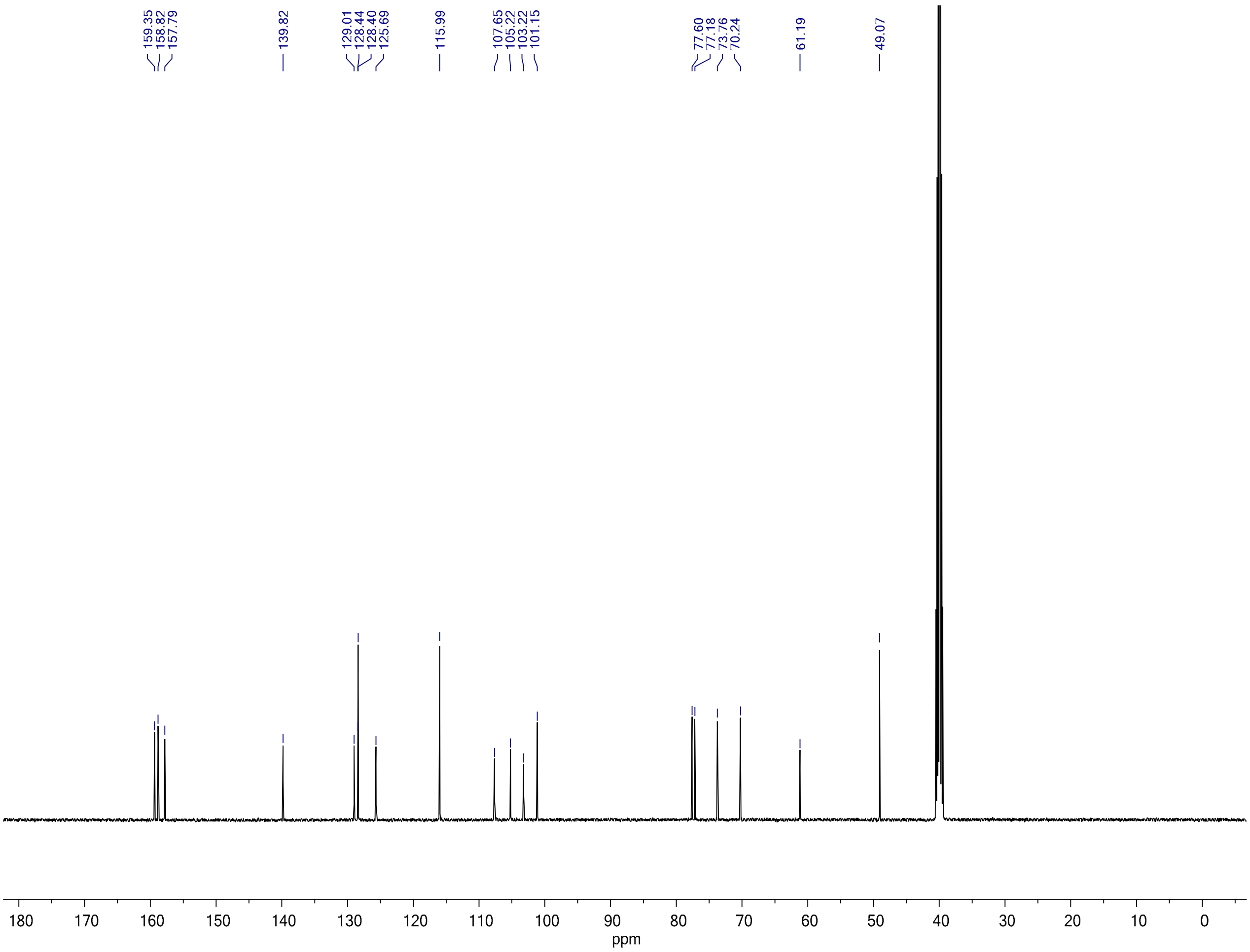

Figure S6. The ^{13}C-NMR spectrum of compound **2** (DMSO-d_6, 125 MHz)

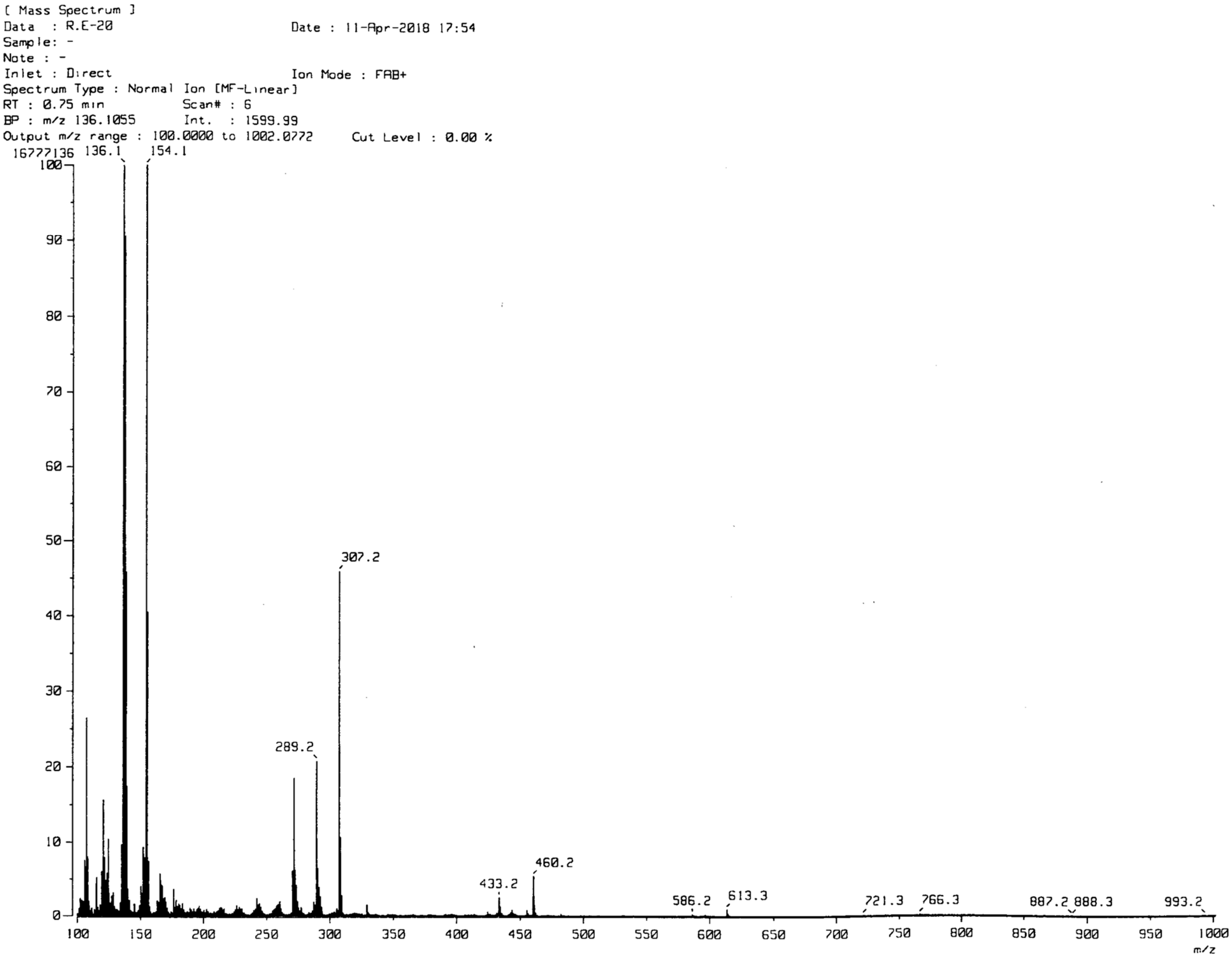

Figure S7. The FAB-MS spectrum of compound **3**

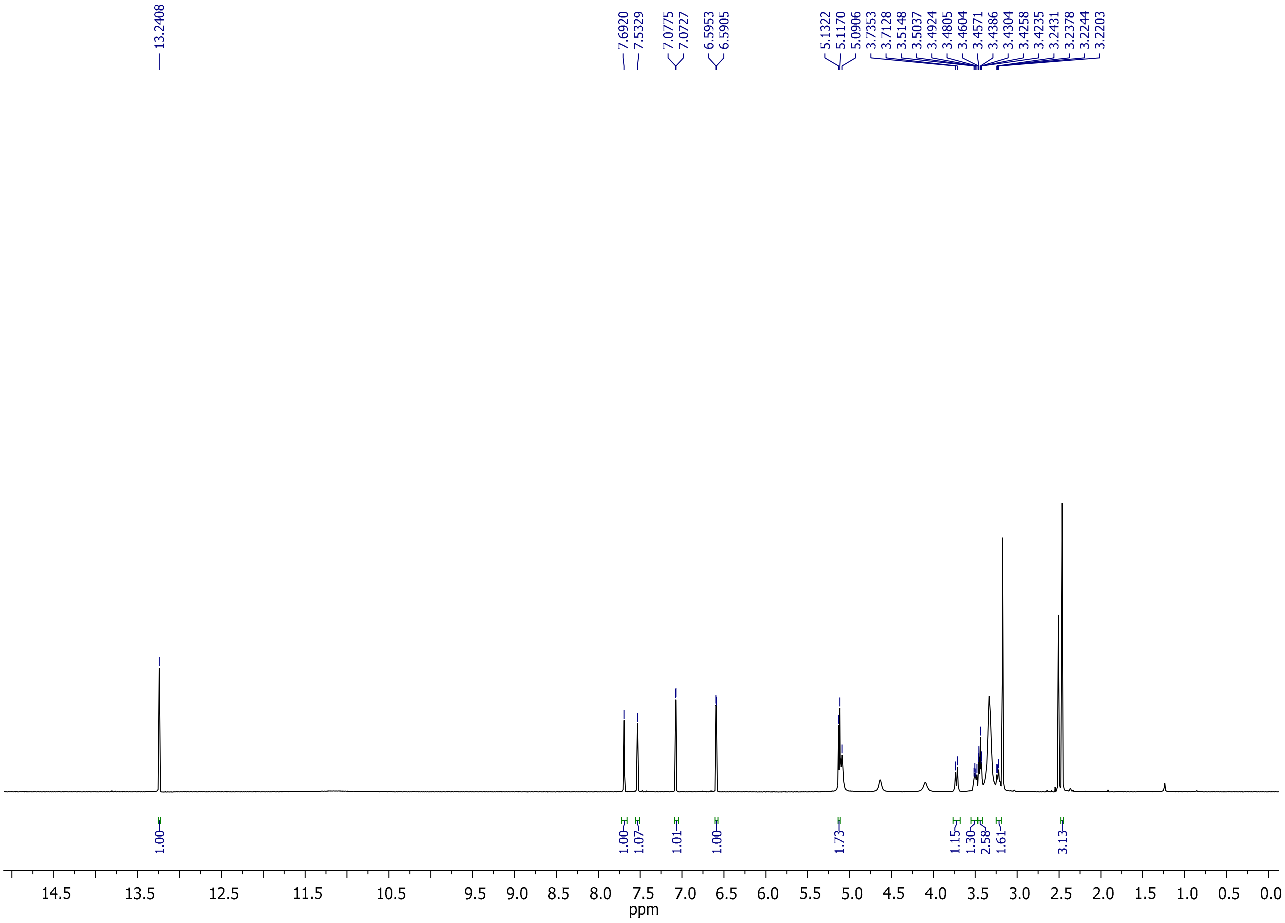

Figure S8. The ^{1}H-NMR spectrum of compound **3** (DMSO-d_6, 500 MHz)

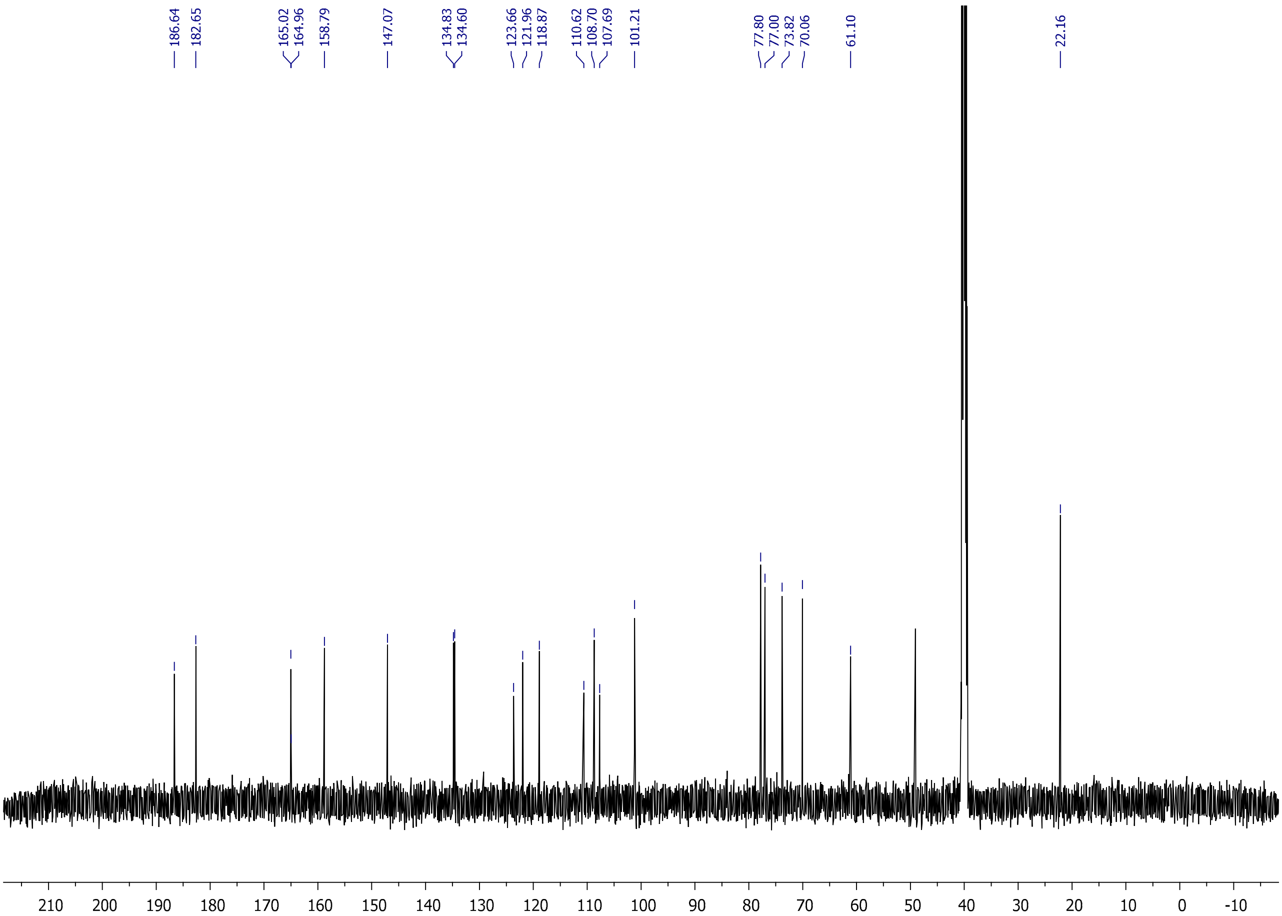

Figure S9. The ^{13}C-NMR spectrum of compound **3** (DMSO-d_6, 125 MHz)

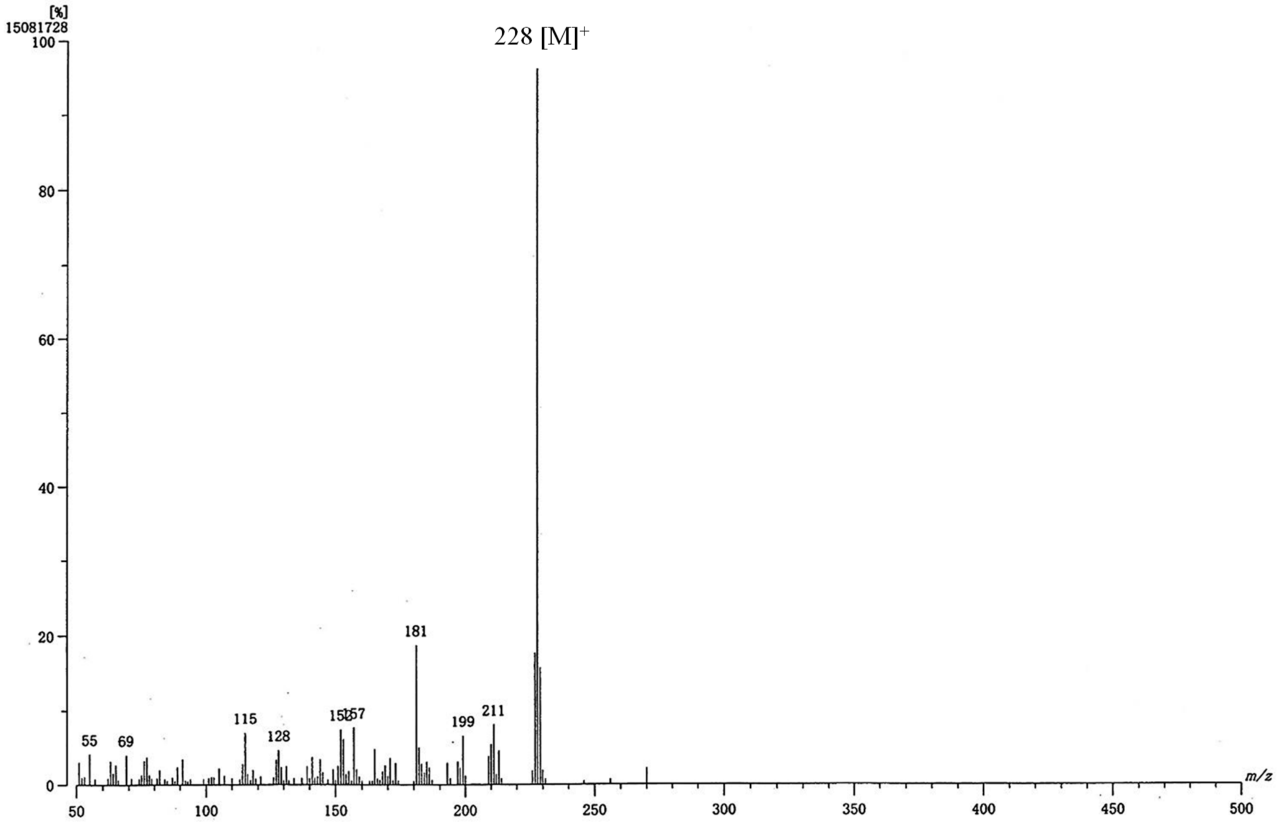

Figure S10. The EI-MS spectrum of compound **4**

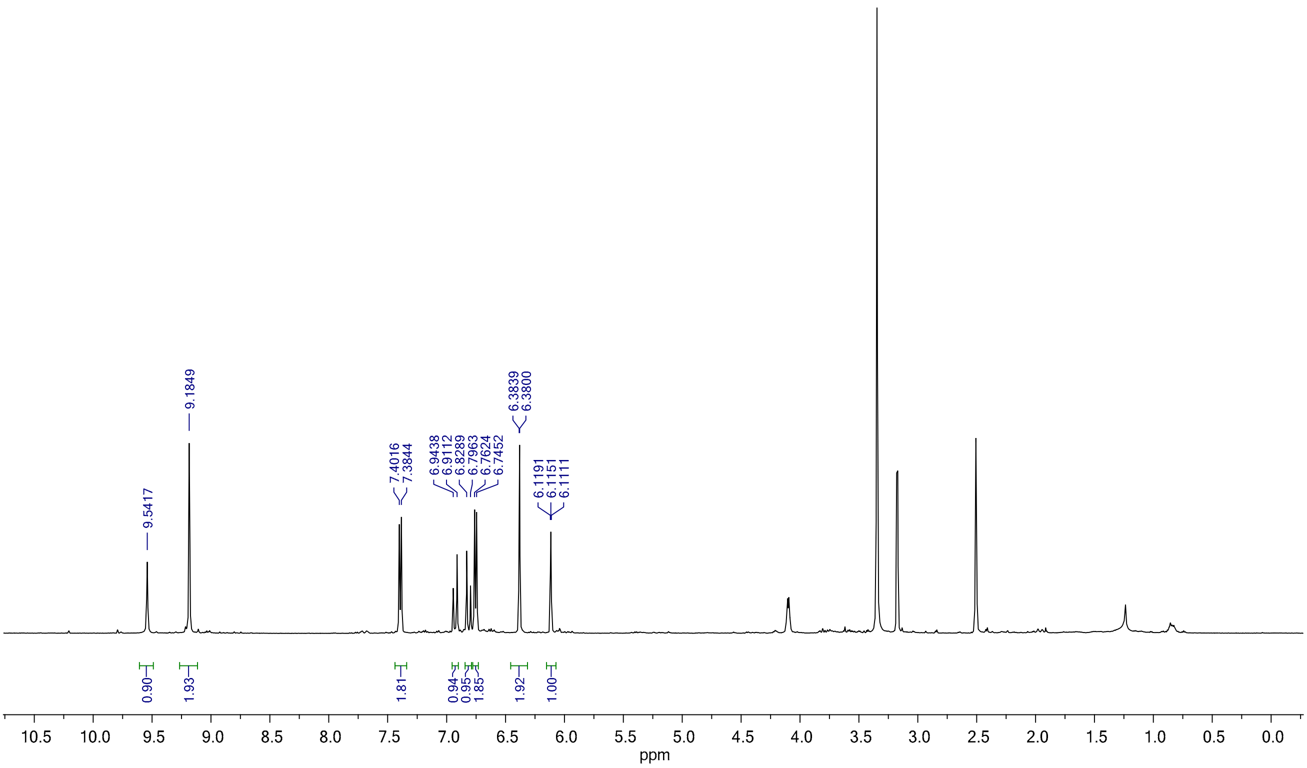

Figure S11. The ^{1}H-NMR spectrum of compound **4** (DMSO-d_6, 500 MHz)

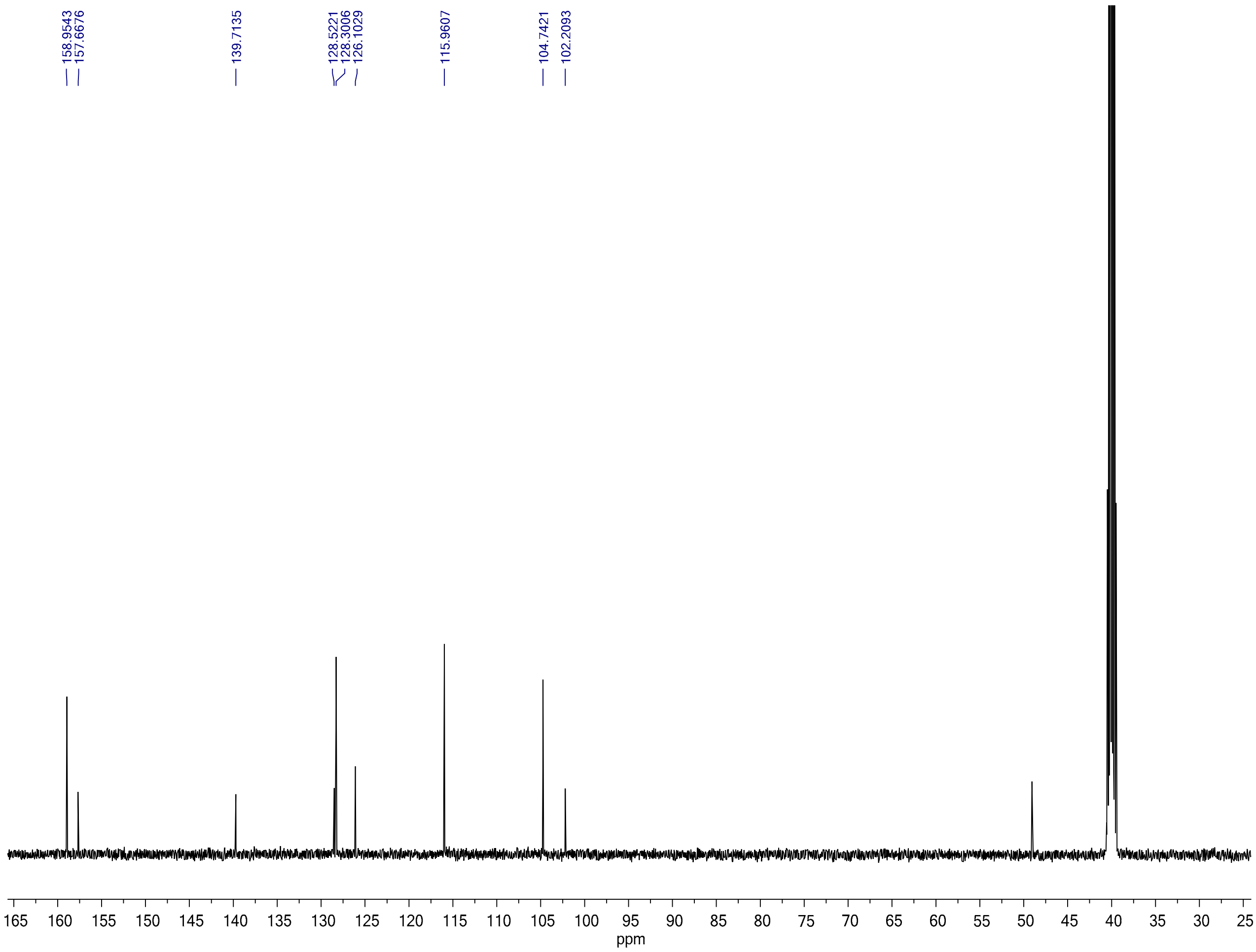

Figure S12. The ^{13}C-NMR spectrum of compound **4** (DMSO-d_6, 125 MHz)

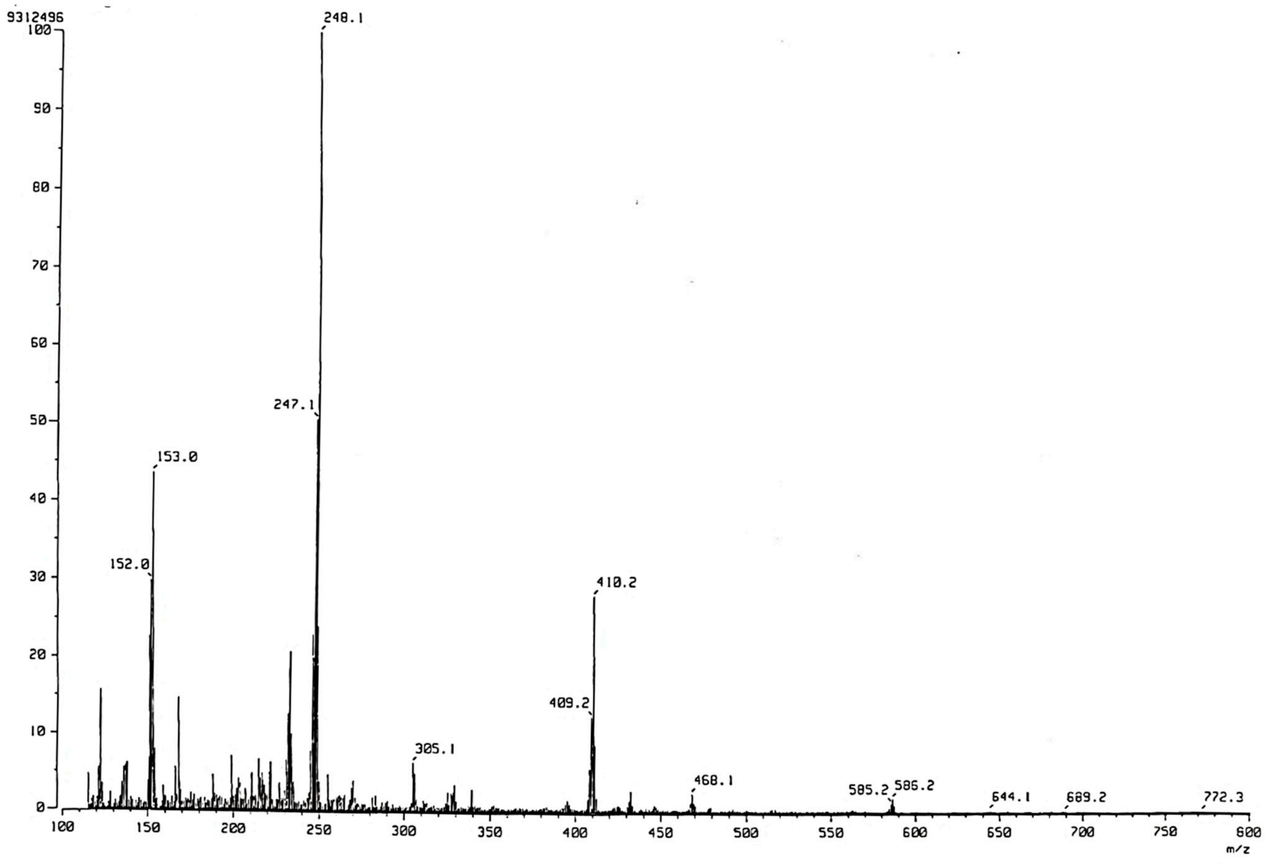

Figure S13. The FAB-MS spectrum of compound **5**

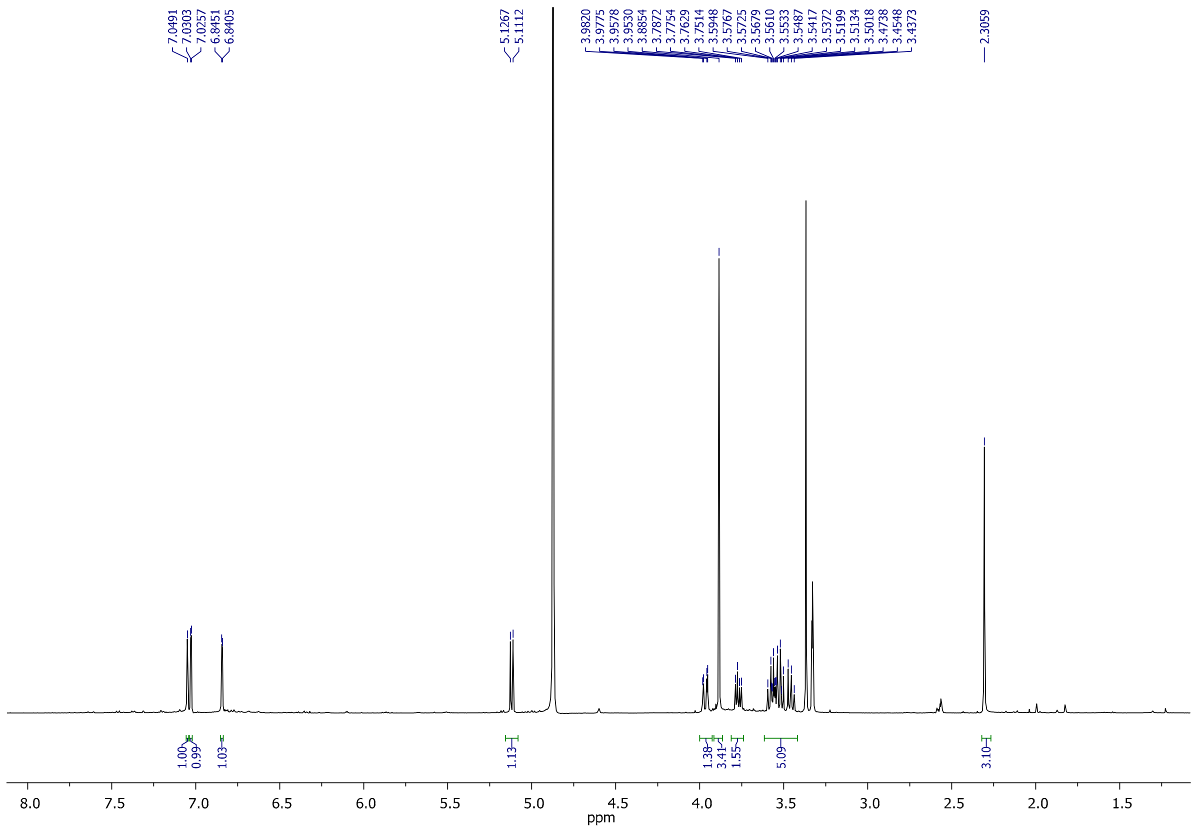

Figure S14. The ^{1}H-NMR spectrum of compound **5** (CD_3OD, 500 MHz)

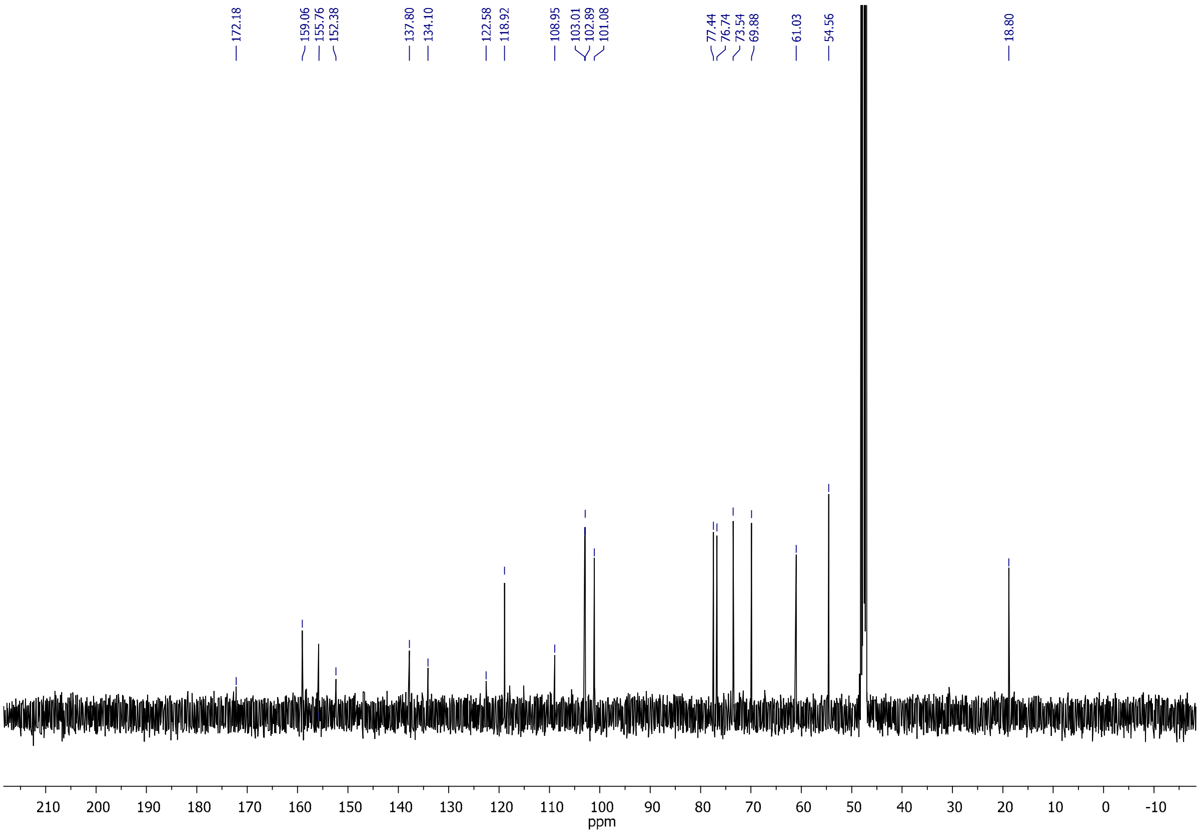

Figure S15. ^{13}C-NMR spectrum of compound **5** **(**CD_3OD, 125 MHz)

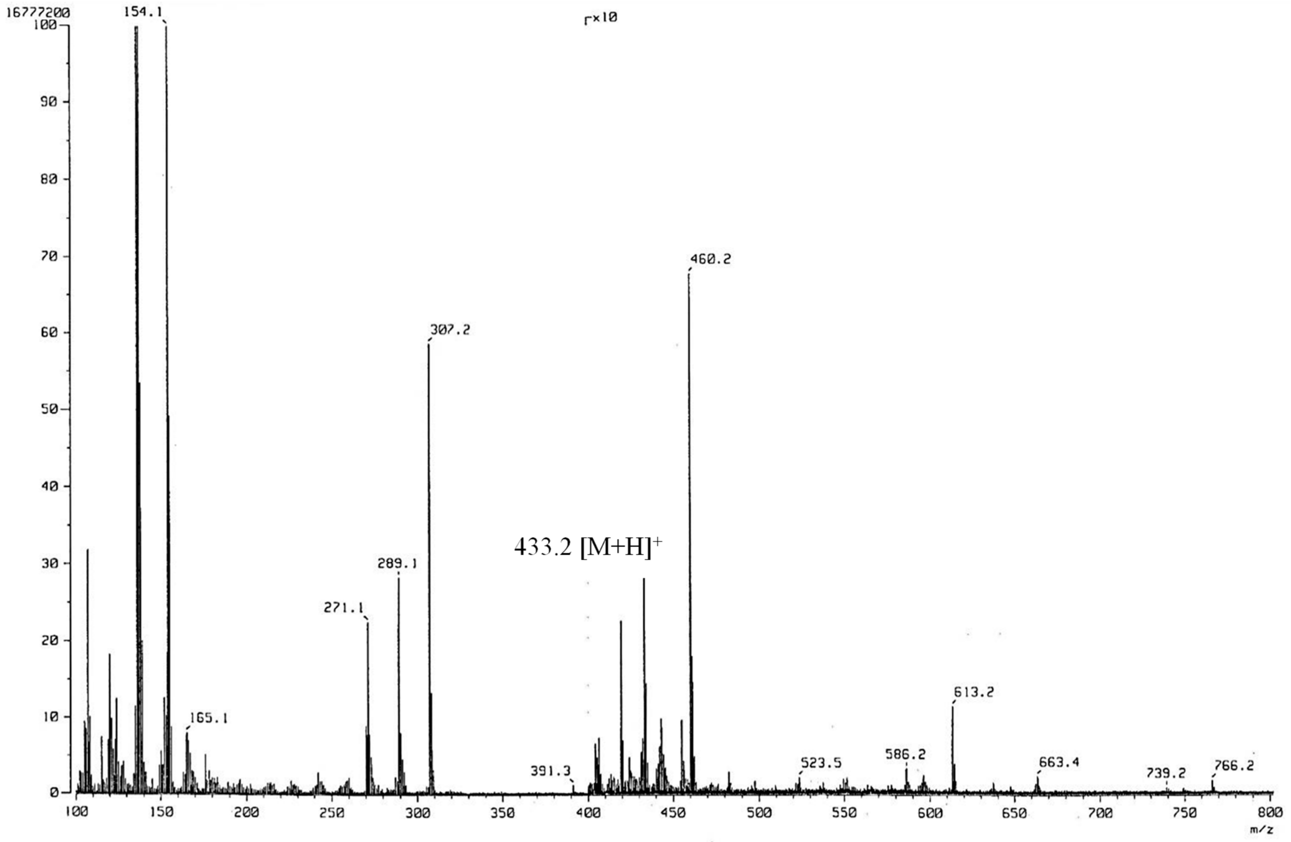

Figure S16. The FAB-MS spectrum of compound **6**

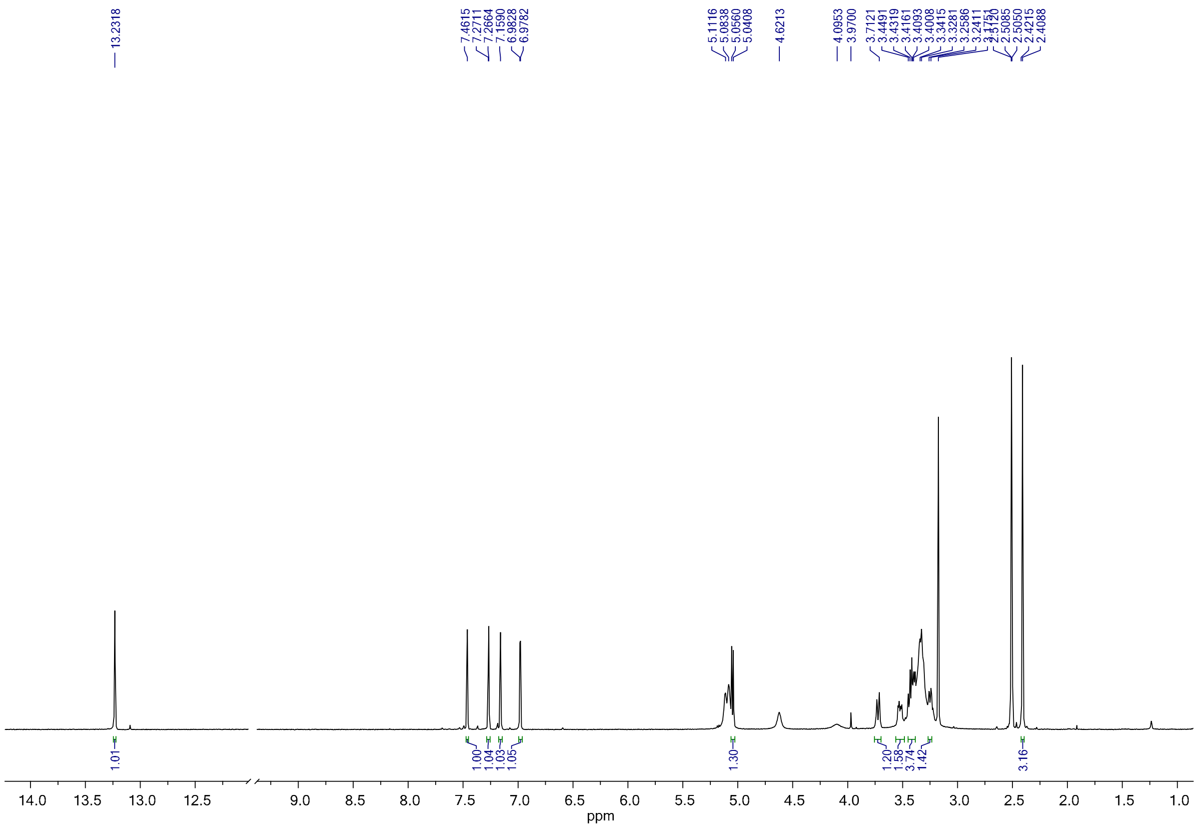

Figure S17. The ^{1}H-NMR spectrum of compound **6** (DMSO-*d*6, 500 MHz)

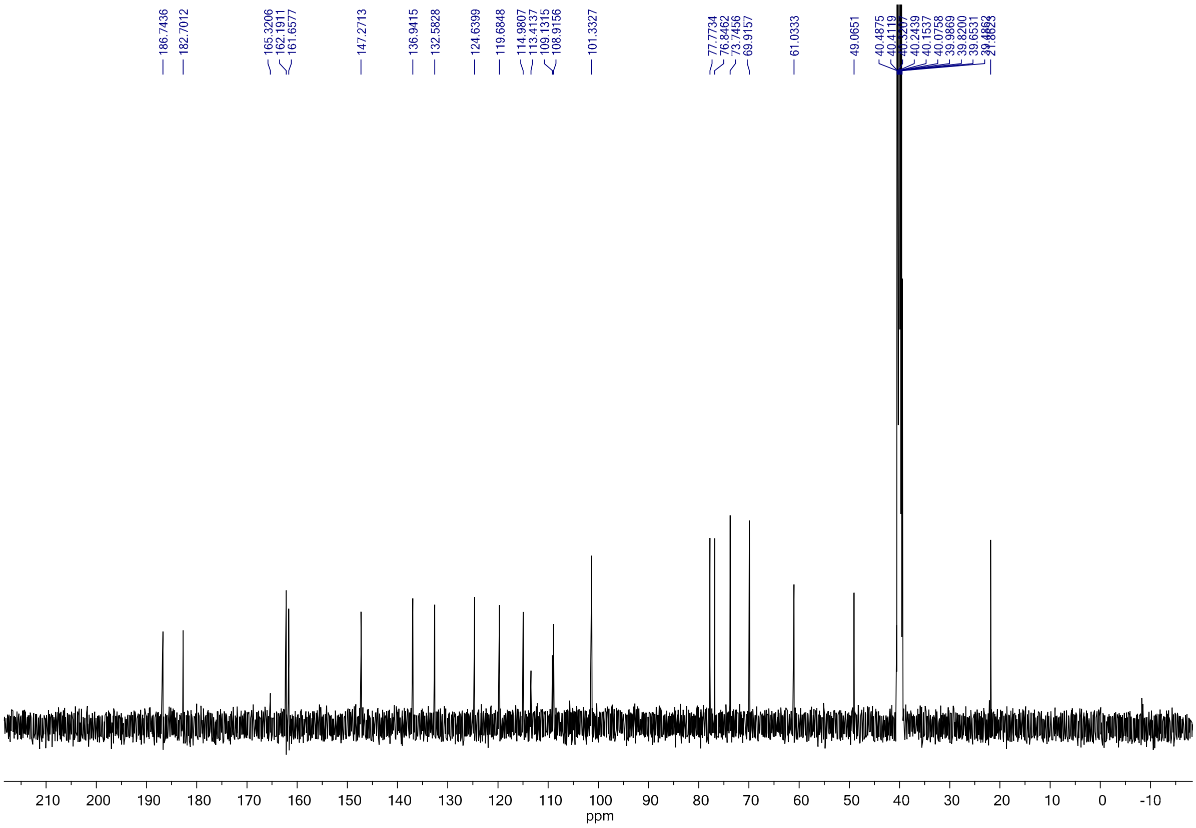

Figure S18. The ^{13}C-NMR spectrum of compound **6** (DMSO-d_6, 125 MHz)

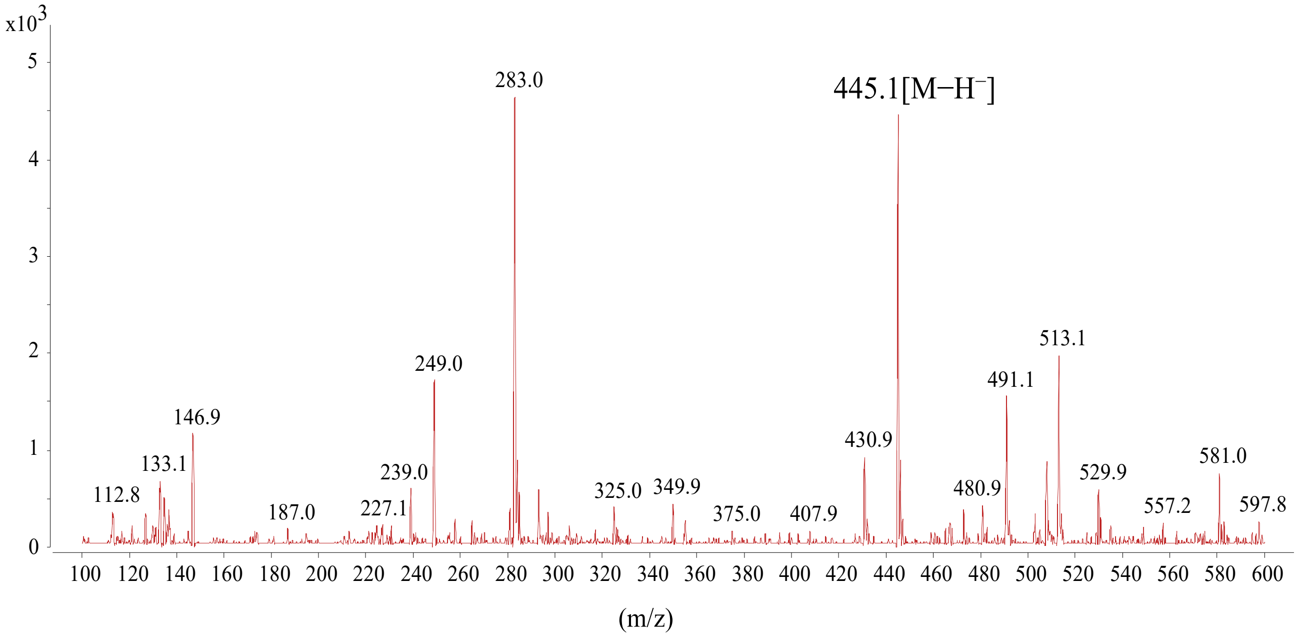

Figure S19. ESI-MS spectrum of compound **7**

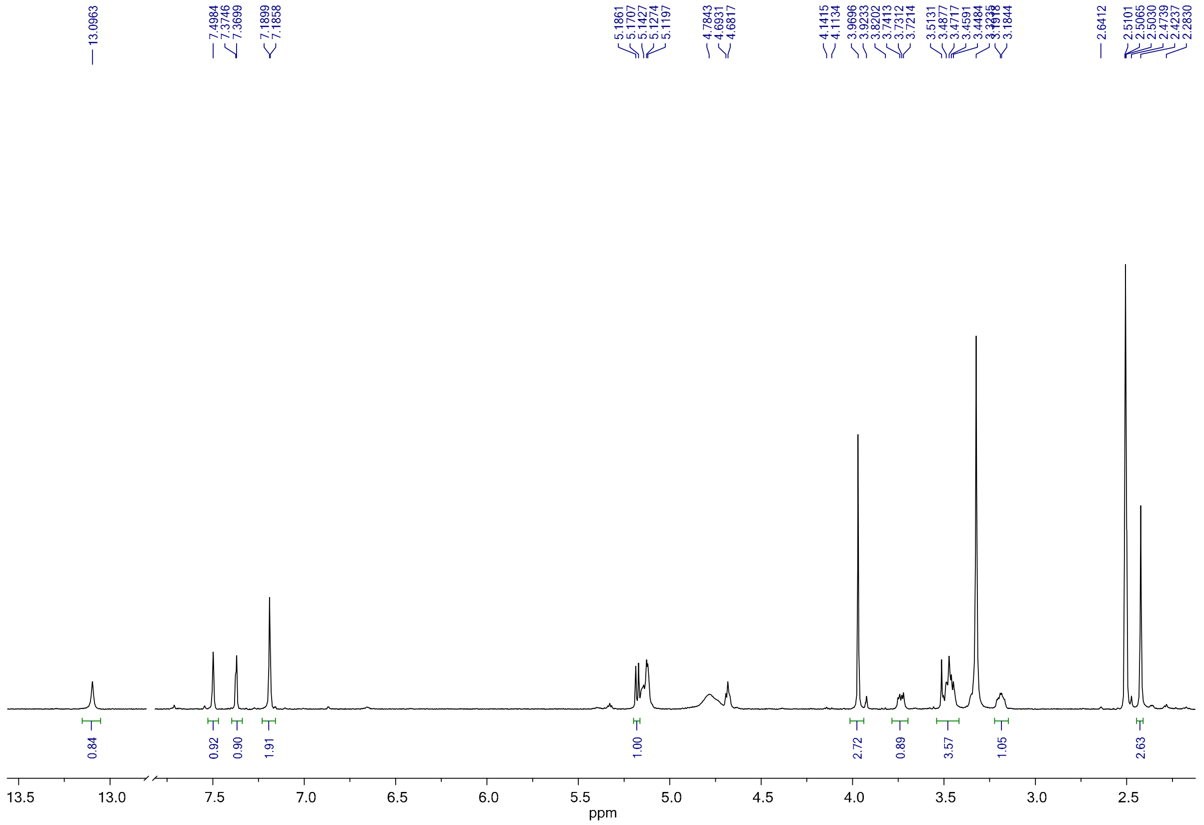

Figure S20. The ^{1}H-NMR spectrum of compound **7** (DMSO-d_6, 500 MHz)

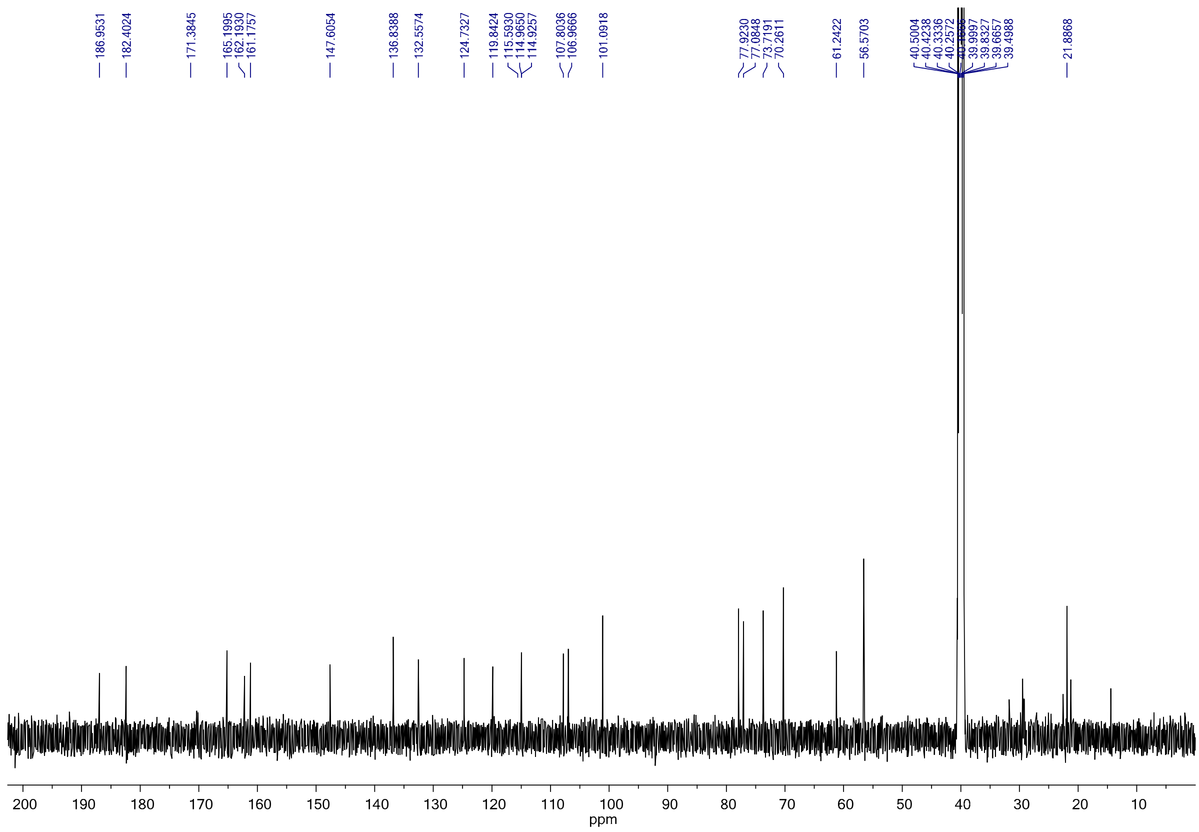

Figure S21. The ^{13}C-NMR spectrum of compound **7** (DMSO-*d*$_6$, 125 MHz)

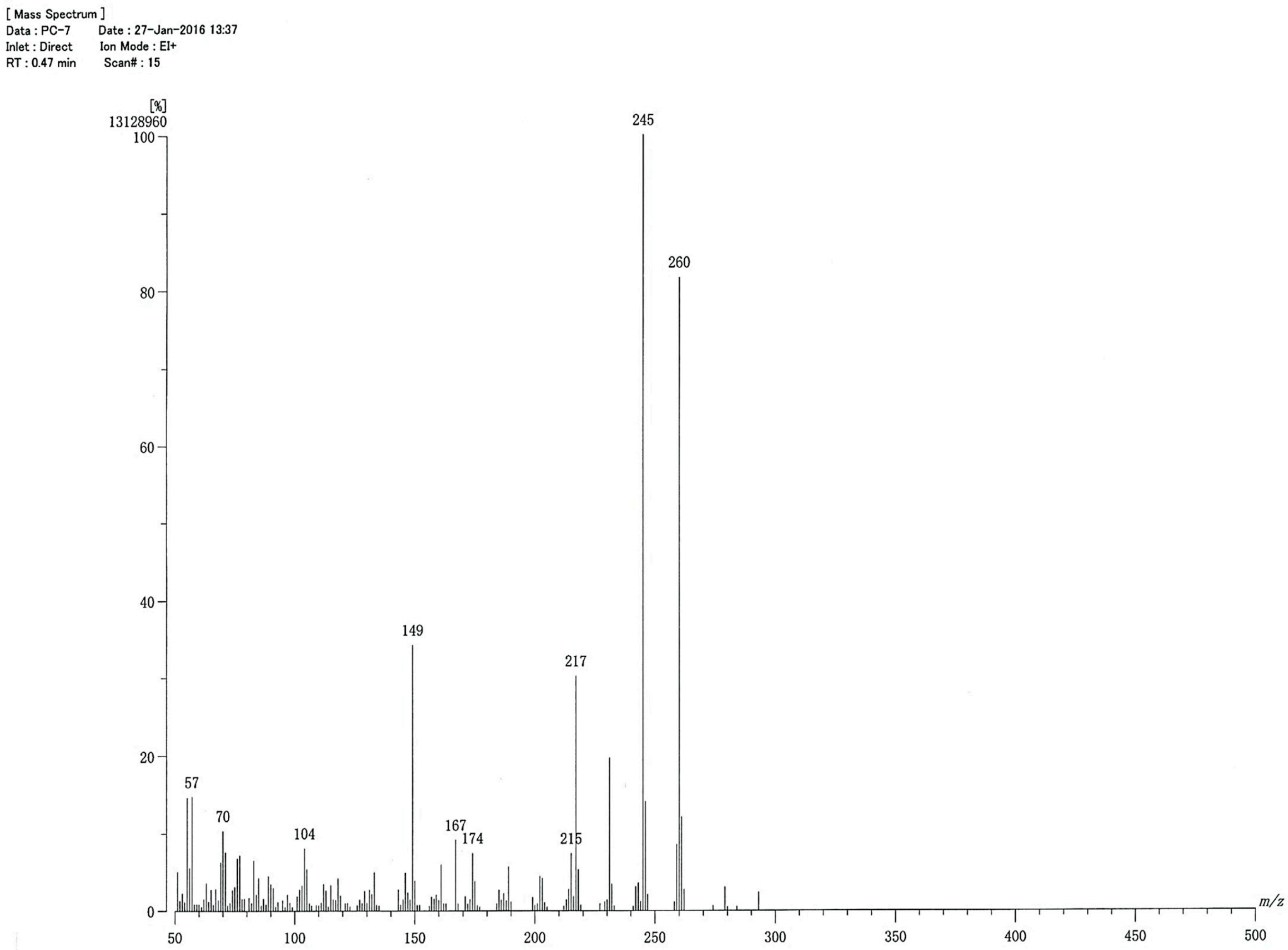

Figure S22. The EI-MS spectrum of compound **8**

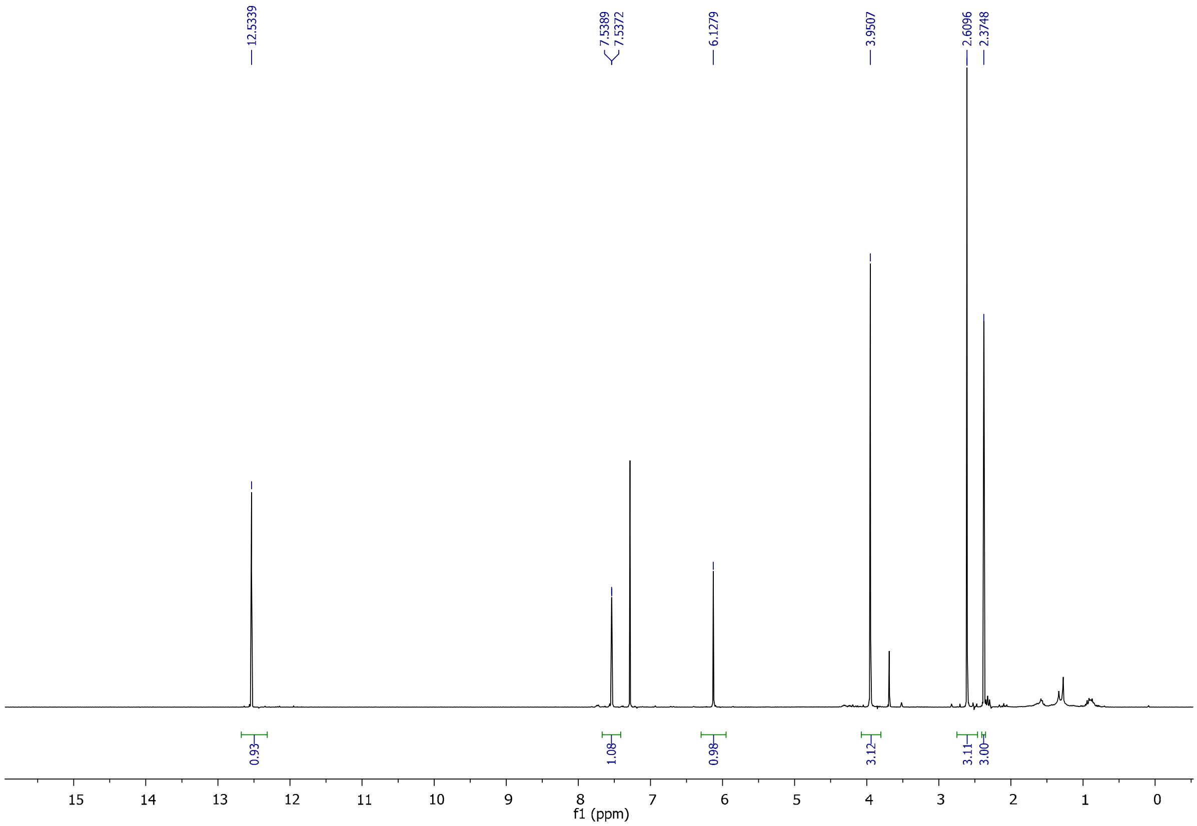

Figure S23. The ^{1}H-NMR spectrum of compound **8** ($CDCl_3$, 300 MHz)

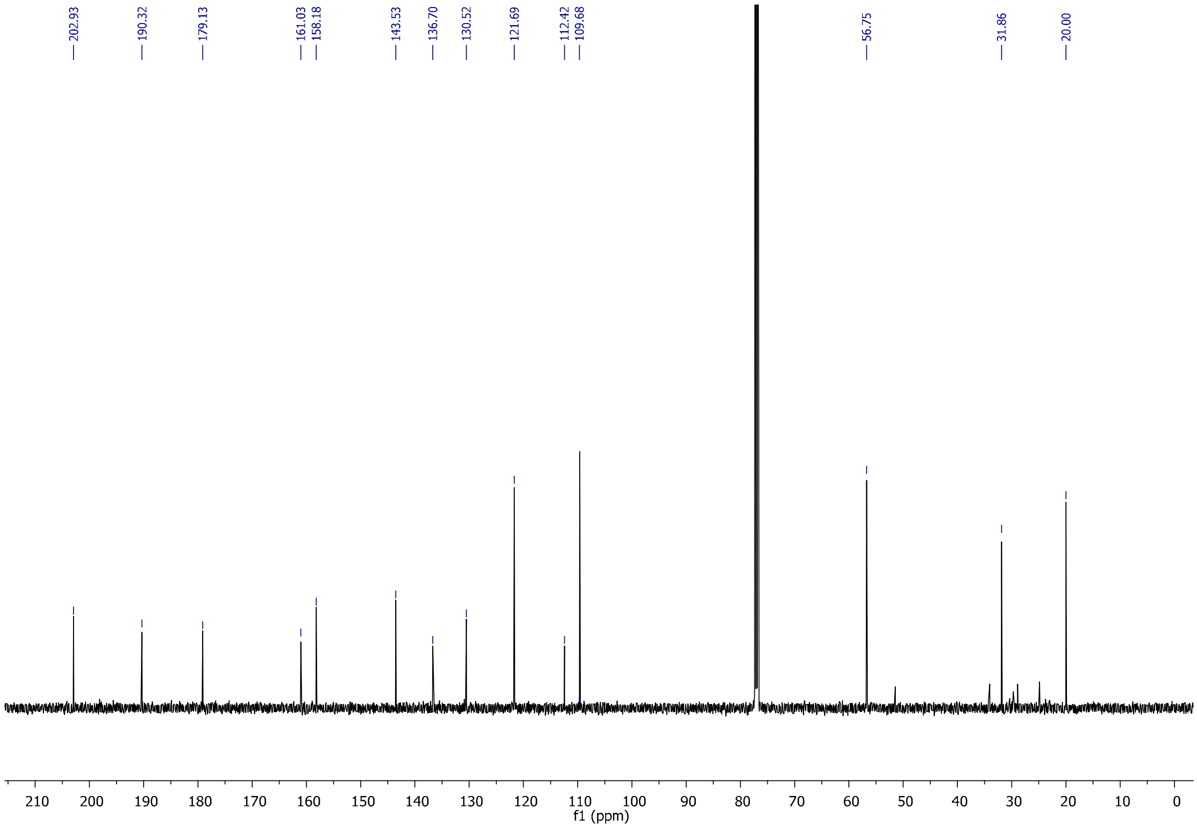

Figure S24. The ^{13}C-NMR spectrum of compound **8** ($CDCl_3$, 125 MHz)

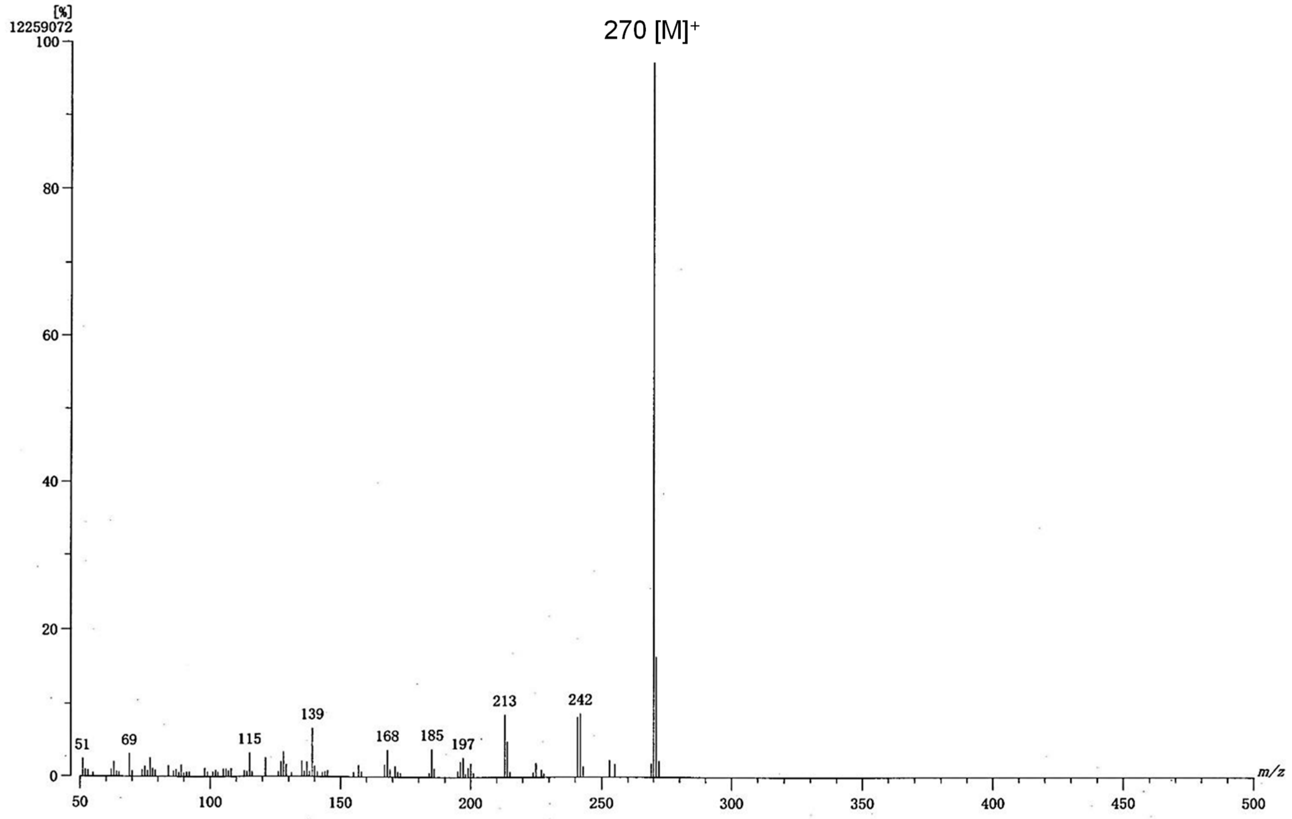

Figure S25. The EI-MS spectrum of compound **9**

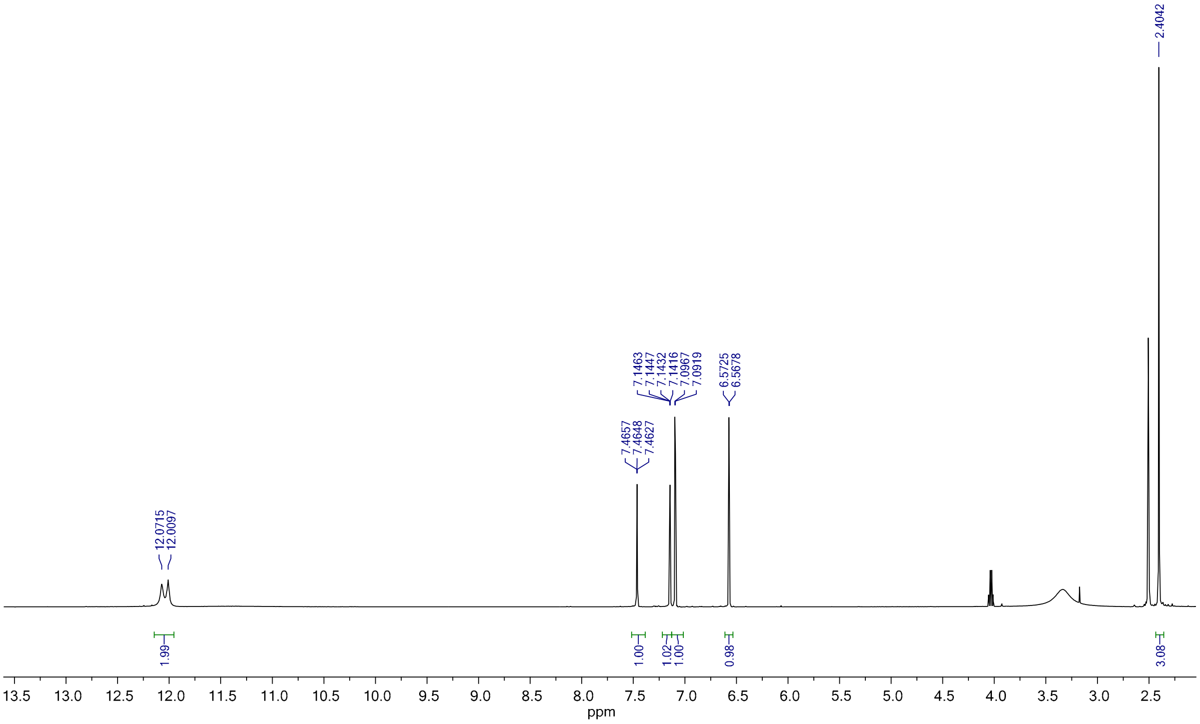

Figure S26. The ^{1}H-NMR spectrum of compound **9** (DMSO-*d*$_6$, 300 MHz)

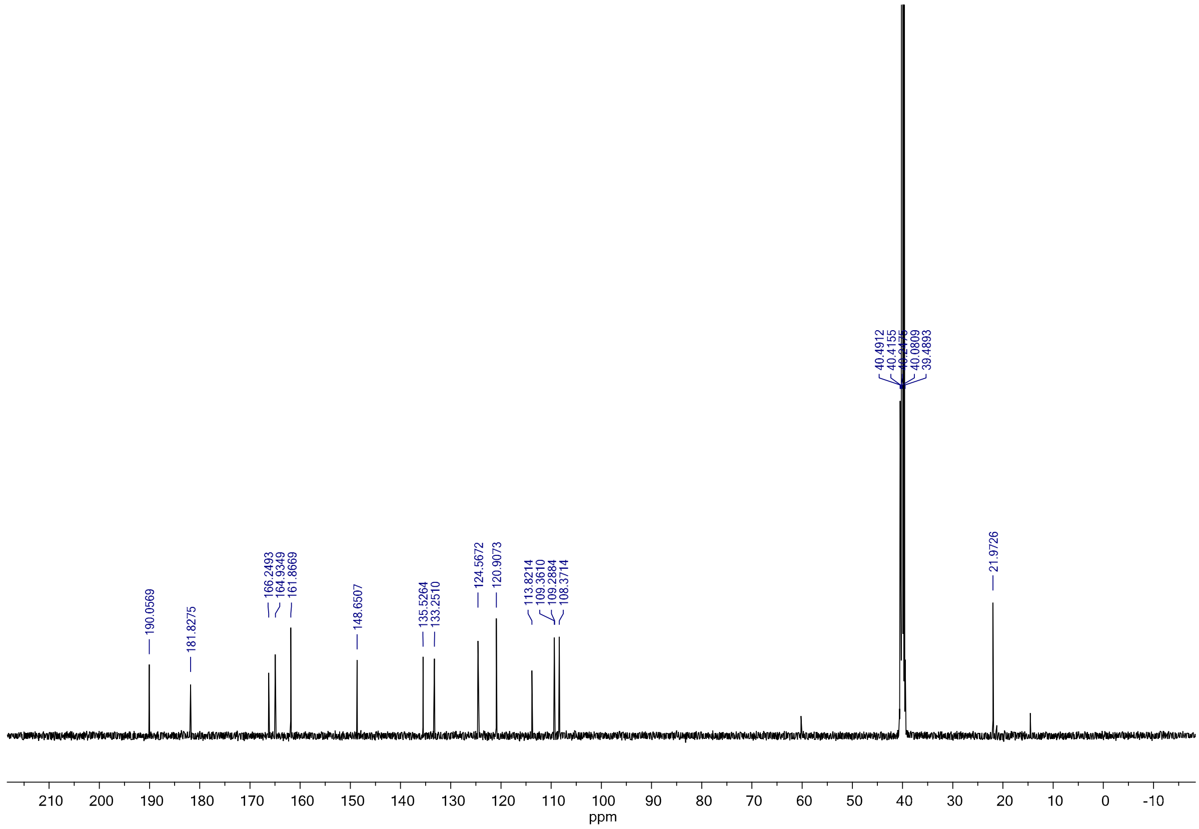

Figure S27. The ^{13}C-NMR spectrum of compound **9** (DMSO-*d*$_6$, 125 MHz)

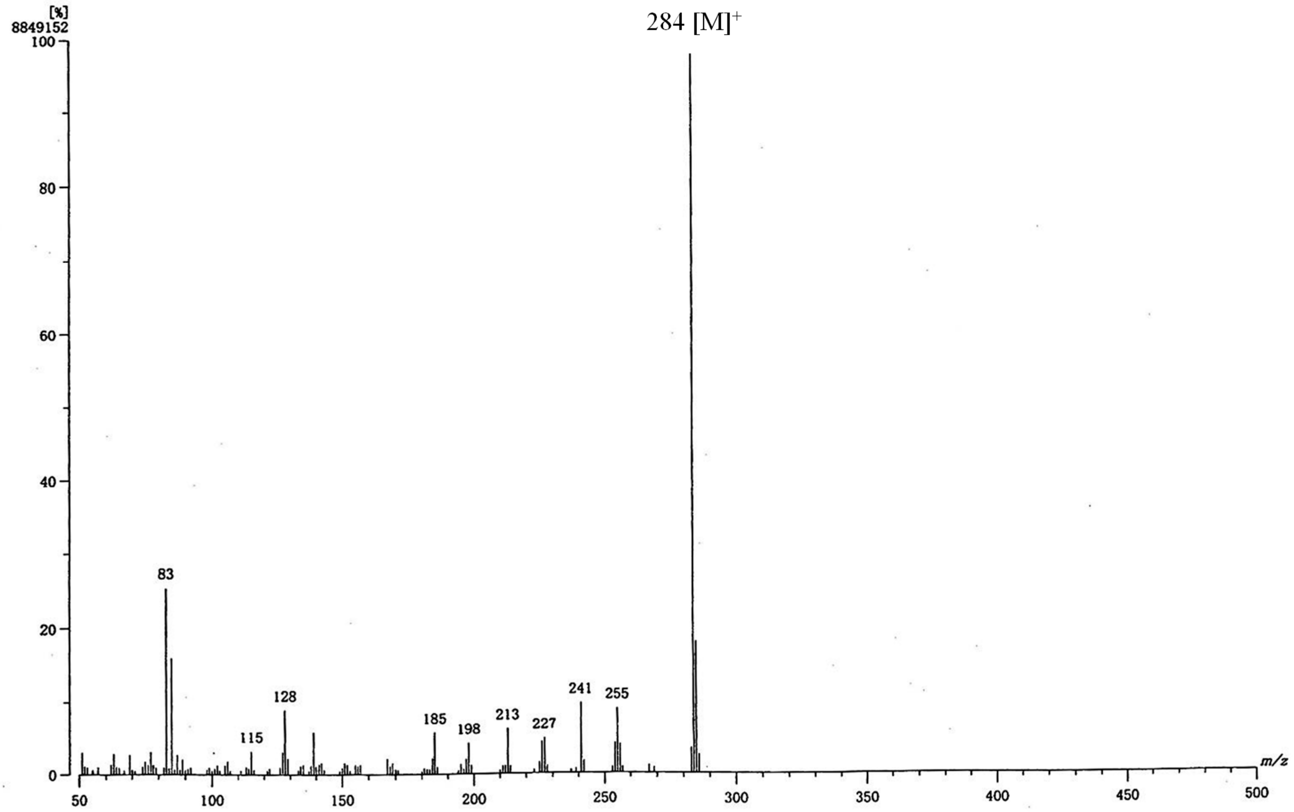

Figure S28. The EI-MS spectrum of compound **10**

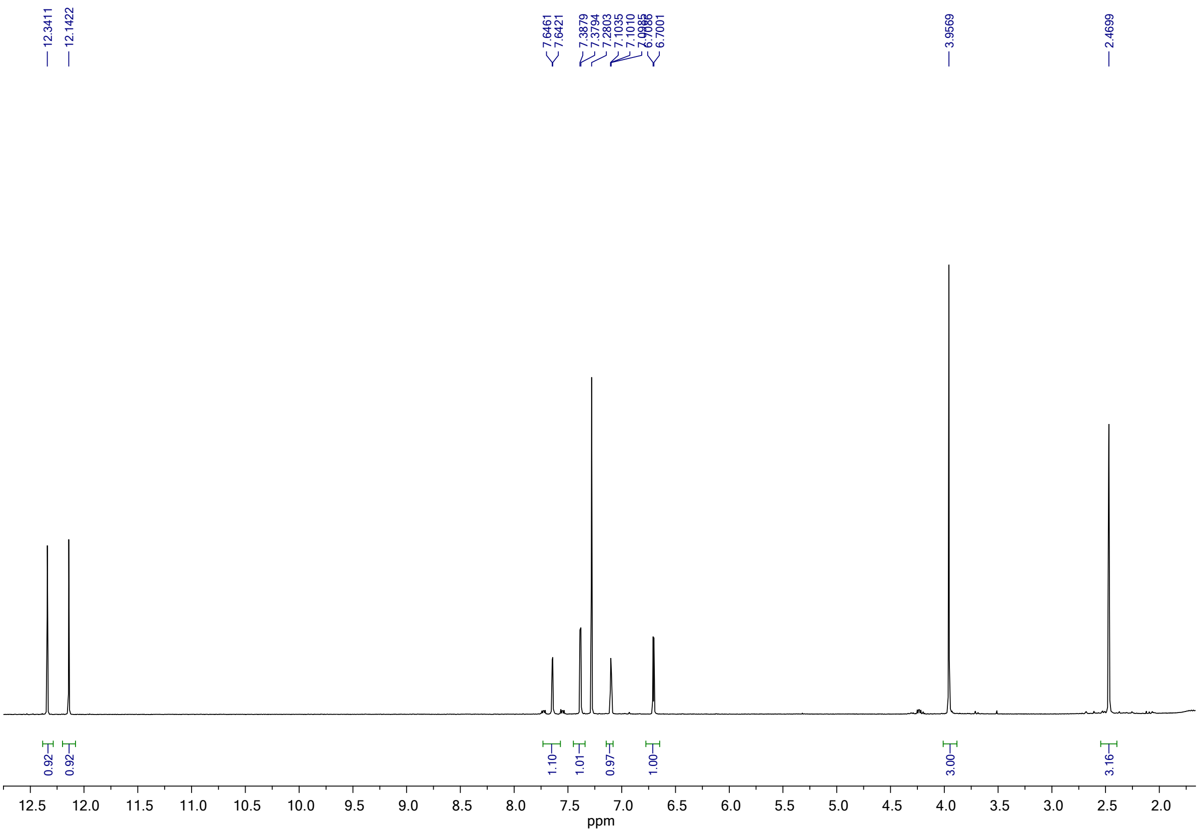

Figure S29. The ^{1}H-NMR spectrum of compound **10** ($CDCl_3$, 300 MHz)

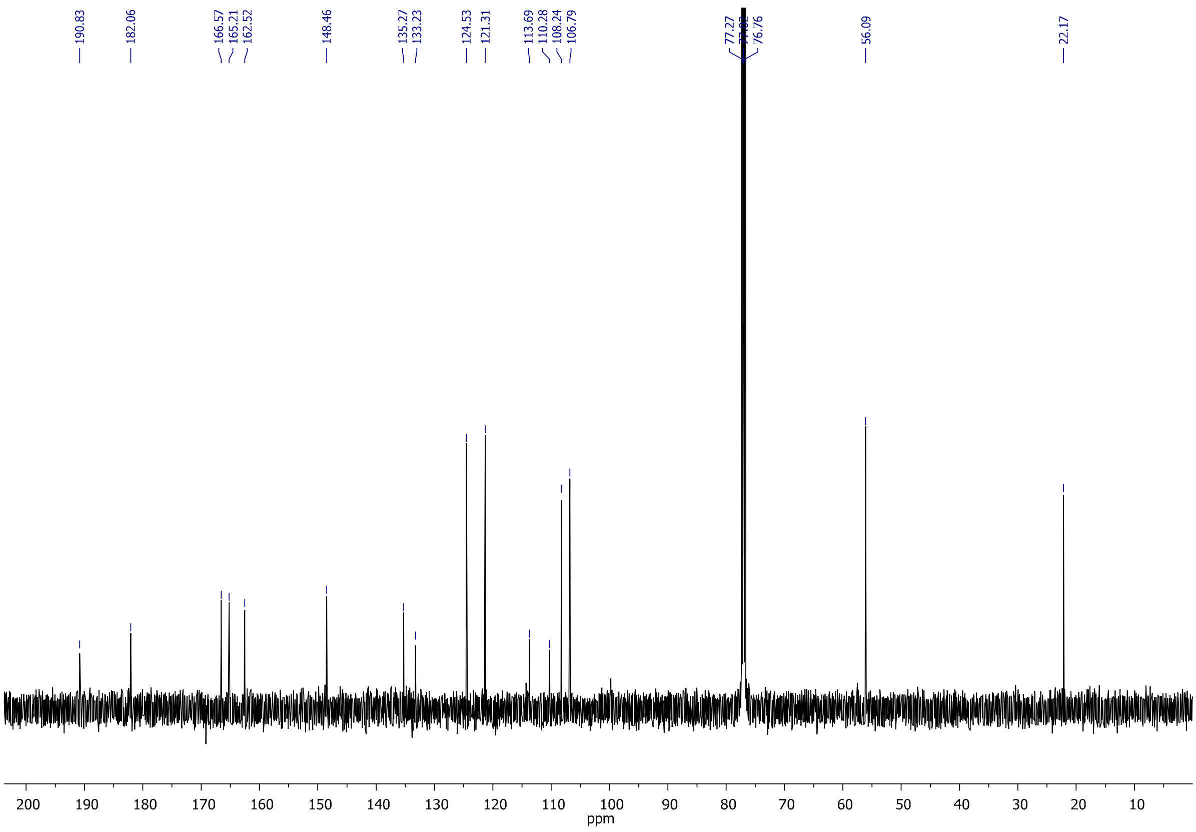

Figure S30. The ^{13}C-NMR spectrum of compound **10** ($CDCl_3$, 125 MHz)

^{1}H and ^{13}C NMR assign data of isolated compounds

Resveratroloside (**1**): Pale white powder; $C_{20}H_{22}O_8$; FAB-MS (*m/z*): 390.2 $[M+H]^+$; ^{1}H-NMR (DMSO-*d*$_6$, 500MHz) *δ*: 9.22 (2H, s, OH-3,5) 7.51 (2H, d, *J* = 8.8 Hz, H-2′,6′), 7.02 (2H, d, *J*=8.8 Hz, H-3′,5′), 7.00 (1H, d, *J* = 16.8 Hz, H-8), 6.93 (1H, d, *J* = 16.8 Hz, H-7), 6.42 (2H, d, *J* = 2.0 Hz, H-2, 6), 6.14 (1H, t, *J* = 2.0 Hz, H-4), 4.89 (1H, d, *J* = 7.4 Hz, H-1″), 3.70 (1H, m, H-a″), 3.48 (1H, m, H-b″), 3.33 (1H, m, H-3″), 3.31 (1H, m, H-5″), 3.26 (1H, m, H-2″), 3.13 (1H, m, H-4″); ^{13}C-NMR (DMSO-*d*$_6$, 125 MHz,) *δ:* 159.0 (C-3,5), 157.5 (C-4′), 139.5 (C-1′), 131.3 (C-1), 128.1 (C-3′,5′), 127.8 (C-8), 127.6 (C-7), 116.9 (C-2′,6′), 104.9 (C-2, 6), 102.5 (C-4), 100.8 (C-1″), 77.5 (C-3″), 77.1 (C-5″), 73.7 (C-2″), 70.2 (C-4″), 61.2 (C-6″).

Polydatin (2): White powder; $C_{20}H_{22}O_8$; FAB-MS (*m/z*): 390.1 $[M]^+$; ^{1}H-NMR (500 MHz, DMSO-*d*$_6$): *δ*: 7.40 (2H, d, *J* = 8.6 Hz, H-2′, 6′), 7.03 (1H, d, *J* = 16.3 Hz, H-b), 6.87 (1H, d, *J* = 16.3 Hz, H-a), 6.76 (2H, d, *J* = 8.6 Hz, H-3′, 5′), 6.74 (1H, br t, H-2), 6.57 (1H, br t, H-6), 6.34 (1H, t, *J* = 2.1 Hz, H-4) 4.81 (1H, d, *J* = 7.6 Hz, glc H-1″), 3.18 ~ 3.49 (glc H-2″ ~ 6″); ^{13}C-NMR (125 MHz, DMSO-*d*$_6$) *δ*: 159.4 (C-3), 158.8 (C-5), 157.8 (C-4′), 139.8 (C-1), 129.0 (C-1′), 128.5 (C-b), 128.4 (C-2′, 6′), 125.7 (C-a), 116.0 (C-3′, 5′), 107.7 (C-6), 105.2 (C-4), 103.2 (C-2), 101.2 (C-1″), 77.6 (C-5″), 77.2 (C-3″), 73.8 (C-2″), 70.2 (C-4″), 61.2 (C-6″)

Emodin-1-*O*-*β*-D-glucoside (**3**): Pale red powder; FAB-MS (*m/z*): 433.2 $[M+H]^+$; ^{1}H-NMR (DMSO-*d*$_6$, 500 MHz) *δ:* 13.24 (1H, s, OH), 7.69 (1H, s, H-4), 7.53 (1H, s, H-2), 7.08 (1H, d, *J* = 2.4 Hz, H-5), 6.59 (1H, d, *J* = 2.4 Hz, H-7), 5.13 (1H, d, *J* = 7.6 Hz, H-1′) 2.50 (3H, s, CH_3), 3.73 (1H, m, H-6a′), 3.51 (1H, m, H-6b′), 3.47 (1H, m, H-5′), 3.43 (1H, m, H-2′), 3.34 (1H, m, H-3′), 3.24 (1H, m, H-4′); ^{13}C-NMR (DMSO-*d*$_6$, 125 MHz) *δ:* 186.6 (C-9), 182.7 (C-10), 165.0 (C-8), 165.0 (C-6), 158.8 (C-1), 147.1 (C-3), 134.8 (C-10a), 134.6 (C-4a), 123.7 (C-2), 122.0 (C-4), 118.9 (C-9a), 110.6 (C-8a), 108.7 (C-7), 107.7 (C-5), 101.2 (C-1′), 77.8 (C-5′), 77.0 (C-3′), 73.8 (C-2′), 70.1 (C-4′), 61.1 (C-6′), 22.2 (CH_3).

***trans*-Resveratrol (4)**: Pale yellow powder; $C_{14}H_{12}O_3$; EI-MS (*m/z*): 228 $[M]^+$; ^{1}H-NMR (500 MHz, DMSO-*d*$_6$) *δ*: 9.54 (4′-OH), 9.18 (3, 5-OH) 7.39 (2H, d, *J* = 8.6 Hz, H-2′, 6′), 6.93 (1H, d, *J* = 16.3 Hz, H-b), 6.81 (1H, d, *J* = 16.3 Hz, H-a), 6.75 (2H, d, *J* = 8.6 Hz, H-3′, 5′), 6.38 (2H, d, *J* = 2.0 Hz, H-2, 6), 6.12 (1H, t, *J* = 2.0 Hz, H-4); ^{13}C-NMR (125 MHz, DMSO-*d*$_6$) *δ*: 159.0 (C-3, 5), 157.7 (C-4′), 139.7 (C-1), 128.5 (C-1′), 128.3 (C-2′, 6′, b), 126.1 (C-a), 116.0 (C-3′, 5′), 104.7 (C-2, 6), 102.2 (C-4)

6-methoxy-3-methyl-1,6,8-trihydroxy-2-naphthic acid-8-*O*-*β*-D-glucoside (5): Pale yellow powder; $C_{19}H_{22}O_{10}$; FAB-MS (*m/z*): 410 $[M]^+$; ^{1}H-NMR (CD_3OD, 500 MHz) *δ*: 7.05 (1H, s, H-4), 7.03 (1H, d, *J* = 2.3 Hz, H-7), 6.84 (1H, d, *J* = 2.3 Hz, H-5), 5.12 (1H, d, *J* = 7.7 Hz, H-1′), 3.89 (3H, s, OCH_3), 2.31 (3H, s, CH_3), 3.97 (1H, m, H-6a'), 3.77 (1H, m, H-6b'), 3.59 (1H, m, H-2'), 3.56 (1H, m, H-5'), 3.55 (1H, m, H-3'), 3.46 (1H, m, H-4'); ^{13}C-NMR (CD_3OD, 125 MHz) *δ*: 171.0 (C-11), 159.1 (C-6), 155.8 (C-8), 152.4 (C-1), 137.8 (C-10), 134.1 (C-3), 122.6 (C-2), 118.9 (C-4), 108.9 (C-9), 103.0 (C-7), 102.9 (C-1'), 101.1 (C-5), 74.4 (C-3'), 76.7 (C-5'), 73.5 (C-2'), 69.9 (C-4'), 61.0 (C-6'), 54.6 (OCH_3), 18.8 (CH_3)

Emodin-8-*O*-*β*-D-glucoside (**6**): Yellowish powder; $C_{21}H_{20}O_{10}$; FAB-MS (*m/z*): 433.2 $[M+H]^+$; ^{1}H-NMR (500 MHz, DMSO-*d*$_6$) δ: 13.2 (1H, br s, OH-1), 7.46 (1H, br s, H-4), 7.27 (1H, d, *J* = 2.4 Hz, H-5), 7.16 (1H, br s, H-2), 6.98 (1H, d, *J* = 2.4 Hz, H-7), 5.05 (1H, d, *J* = 7.6 Hz, H-1′), 3.72 (1H, m, H-6′a), 3.52 (1H, m, H-6′b), 3.43 (1H, m, H-2′), 3.39 (1H, m, H-5′), 3.33 (1H, m, H-3′), 3.24 (1H, m, H-4′) 2.41 (3H, s, 3-CH_3); ^{13}C-NMR (125

MHz, DMSO-d_6) δ: 186.7 (C-9), 182.7 (C-10), 165.5 (C-6), 162.2 (C-1), 161.7 (C-8), 147.3 (C-3), 136.9 (C-10a), 132.6 (C-6), 124.6 (C-2), 119.7 (C-4), 115.00 (C-9a), 113.4 (C-8), 109.1 (C-5), 108.9 (C-7), 101.3 (C-1′), 77.8 (C-5′), 76.9 (C-3′), 73.8 (C-2′), 69.9 (C-4′), 61.0 (C-6′), 21.86 (3-CH_3)

Physcion-8-*O*-*β*-D-glucoside (**7**): Yellowish powder ; $C_{22}H_{22}O_{10}$; ESI-MS (*m/z*): 445.1 $[M-H]^-$; ^{1}H-NMR (500 MHz, DMSO-d_6) δ: 13.10 (1-OH), 7.50 (1H, br s, H-4), 7.37 (1H, br d, H-5), 7.19 (2H, br d, H-2,7), 5.18 (1H, d, *J* = 7.7 Hz, H-1′), 3.51 ~ 3.19 (glc-H 2′ ~ 6′), 3.97 (3H, s, 6-OCH_3), 2.41 (3H, s, 3-CH_3); ^{13}C-NMR (125 MHz, DMSO-d_6) δ: 187.0 (C-9), 182.4 (C-10), 165.2 (C-6), 162.2 (C-8), 161.2 (C-1), 147.6 (C-3), 136.8 (C-10a), 132.6 (C-4a), 124.6 (C-2), 119.8 (C-4), 115.0 (C-8a), 114.9 (C-9a), 108.7 (C-7), 107.0 (C-5), 101.1 (C-1′), 77.9 (C-5′), 77.1 (C-3′), 73.7 (C-2′), 70.3 (C-4′), 61.2 (C-6′), 56.6 (6-OCH_3), 21.9 (3-CH_3)

2-Methoxy-6-acetyl-7-methyljuglone (**8**): Red needles; $C_{14}H_{12}O_5$; EI-MS (*m/z*): 260 $[M]^+$; ^{1}H-NMR ($CDCl_3$, 300 MHz) *δ*: 12.53 (1H, s, 5-OH), 7.54 (1H, s, H-8), 6.13 (1H, s, H-3), 3.95 (3H, s, 2-OCH_3), 2.61 (3H, s, 6-$COCH_3$), 2.37 (3H, s, 7-CH_3); ^{13}C-NMR ($CDCl_3$, 125 MHz) *δ*: 202.9 ($\underline{C}OCH_3$), 190.3 (C-4), 179.1 (C-1), 161.0 (C-2), 158.1 (C-5), 143.5 (C-7), 136.7 (C-9), 130.5 (C-6), 121.7 (C-8), 112.4 (C-10), 109.7 (C-3), 56.8 (2-OCH_3), 31.9 (6-$CO\underline{CH}_3$), 20.0 (7-CH_3).

Emodin (**9**): Orange needles; $C_{15}H_{10}O_5$; EI-MS (*m/z*): 270 $[M]^+$; ^{1}H-NMR (300 MHz, DMSO-d_6) *δ*: 12.07 (1H, s, 1-OH), 12.01 (1H, s, 8-OH), 7.46 (1H, br d, H-4), 7.15 (1H, br d, H-2), 7.09 (1H, d, *J* = 2.4 Hz, H-5), 6.57 (1H, d, *J* = 2.4 Hz, H-7), 2.47 (3H, s, 3-CH_3); ^{13}C-NMR (125 MHz, DMSO-d_6) *δ*: 190.6 (C-9), 181.8 (C-10), 166.3 (C-8), 164.9 (C-1), 161.9 (C-6), 148.7 (C-3), 135.5 (C-10a), 133.3 (C-4a), 124.6 (C-4), 120.9 (C-2), 113.8 (C-9a), 109.4 (C-5), 109.3 (C-8a), 108.4 (C-7), 22.2 (3-CH_3)

Physcion (**10**): Yellowish powder; $C_{16}H_{12}O_5$; EI-MS (*m/z*): 284 $[M]^+$; ^{1}H-NMR (300 MHz, $CDCl_3$) *δ*: 12.34 (1H, s, 1-OH), 12.14 (1H, s, 8-OH), 7.64 (1H, d, *J* = 1.2 Hz, H-4), 7.38 (1H, d, *J* = 2.6 Hz, H-5), 7.10 (1H, d, *J* = 1.2 Hz, H-2), 6.70 (1H, d, *J* = 2.6 Hz, H-7), 3.96 (3H, s, 6-OCH_3), 2.47 (3H, s, 3-CH_3); ^{13}C-NMR (125 MHz, $CDCl_3$) *δ*: 190.8 (C-9), 182.1 (C-10), 166.6 (C-8), 165.2 (C-1), 162.5 (C-6), 148.5 (C-3), 135.3 (C-10a), 133.2 (C-4a), 124.5 (C-4), 121.3 (C-2), 113.7 (C-9a), 110.3 (C-8a), 108.2 (C-5), 106.8 (C-7), 56.1 (6-OCH_3), 22.2 (3-CH_3)
